# Evaluation the role of *Luteibacter pinisoli* DP2-30 in mitigating pine wilt disease caused by *Bursaphelenchus xylophilus* through modulation of host microbiome

**DOI:** 10.3389/fpls.2025.1515506

**Published:** 2025-03-05

**Authors:** Waqar Ahmed, Wenhua Ye, Jidong Pan, Songsong Liu, Wenxia Ji, Shun Zhou, Fusheng Wang, Zhiguang Li, Mohamed Mohany, Xinrong Wang

**Affiliations:** ^1^ Guangdong Province Key Laboratory of Microbial Signals and Disease Control, College of Plant Protection, South China Agricultural University, Guangzhou, Guangdong, China; ^2^ Department of Pharmacology and Toxicology, College of Pharmacy, King Saud University, Riyadh, Saudi Arabia

**Keywords:** *Bursaphelenchus xylophilus*, *Luteibacter pinisoli* DP2-30, biocontrol, host microbiome manipulation, forest health

## Abstract

**Background and aim:**

Pine wilt disease (PWD), caused by the pine wood nematode (PWN) *Bursaphelenchus xylophilus*, poses a significant threat to pine forests worldwide. This study aimed to isolate bacterial strains from the rhizosphere of healthy *Pinus massoniana* and elucidate their biocontrol potential in mitigating PWD through direct nematicidal activity and manipulation of host microbiome.

**Results:**

We successfully isolated the rhizobacterium strain DP2-30 from rhizosphere of healthy pine plants, which was identified as *Luteibacter pinisoli* on the basis of morphological, biochemical, and molecular analyses. The fermentation filtrates of strain DP2-30 displayed direct nematicidal activity of >95% (corrected mortality rate) on PWN after 48 hours of treatment. The fermentation broth and filtrates of strain DP2-30 significantly inhibited PWN egg hatching by 49.38% and 43.05%, respectively. Additionally, root drenching of strain DP2-30 fermentation broth significantly reduced PWD severity in pine seedlings (2 years old), with a control effect of 62.50%. Microbiome analyses revealed significant variations in the diversity, structure, and relative abundance of bacterial and fungal communities of pine plants combined treated with DP2-30 and PWN (T2), solely treated with PWN (T1), and control (treated with water). Bacterial phyla, Proteobacteria, Actinobacteriota, Chloroflexi, Acidobacteriota, and Armatimonadota and fungal phyla Ascomycota, Basidiomycota and Mortierellomycota were dominant in the all root and stem samples. The application of *L. pinisoli* DP2-30 significantly increased the relative abundance of the family Rhodanobacteraceae in the roots and stems of pine seedlings. Additionally, intra-kingdom co-occurrence network analysis revealed reduced complexity in the bacterial networks but increased complexity in the fungal networks of treated plants, suggesting enhanced functional redundancy and ecosystem stability.

**Conclusions:**

Overall, this study highlights the potential of *L. pinisoli* DP2-30 as an effective biocontrol agent against PWD by directly killing PWN and manipulating the host microbiota.

## 1 Introduction

Plant-parasitic nematodes (PPNs) are economically significant pests that negatively impact crop yield (Ayaz et al., 2021). To date, ~4100 species of PPNs have been documented in the literature, resulting in annually economic losses exceeding $150 billion (USD), globally (Diyapoglu et al., 2022). *Bursaphelenchus xylophilus*, the pine wood nematode (PWN), is a highly destructive pathogen responsible for causing pine wilt disease (PWD), which poses a substantial threat to pine forests worldwide (Kamaruzzaman et al., 2024) and ranks as the sixth most economically important PPN (Jones et al., 2013). PWN is originally native to North America, however it has been subsequently spread to numerous regions of Asia and Europe, resulting in significant ecological and economic losses (Dou and Yan, 2022). Insect vectors, usually longhorn beetles of the genus *Monochamus*, transmit and introduce the PWN into healthy pine trees from infected pine trees during oviposition or feeding (Akbulut and Stamps, 2012). Upon entering the susceptible host, the PWN undergoes fast reproduction and dissemination throughout the tree’s vascular system, resulting in symptoms of wilting and eventually leading to the death of the whole tree (Modesto et al., 2022). Disease development is particularly severe in vulnerable pine species under favorable climatic conditions such as drought stress (Estorninho et al., 2022) and elevated temperatures (Pimentel and Ayres, 2018). Considering the far-reaching importance of pine forests for biodiversity preservation, ecological services, and timber supply the management of PWD and the mitigation of PWN effects on forest ecosystems have become the main objectives of researchers worldwide.

Currently, PWD management strategies focus on a combination of monitoring, early detection, and rapid response approaches (Chen et al., 2023). Integrated pest management (IPM) approaches such as cultural practices (improving tree health by appropriate watering, mulching, and pruning), biological control approaches, and chemical applications (insecticides and nematicides) are widely adopted to mitigate PWD incidence (Kim et al., 2020a). Efforts are being made in certain regions to decrease the population of pine sawyer beetles (*Monochamus* spp.), which serve as carriers for the PWN (He et al., 2020; Kim et al., 2020b). Additionally, there is an increasing focus on utilizing pine species (*Pinus pinea* and *P. radiata*) having resistant to PWN for reforestation and landscaping to mitigate incidence of PWD more effectively (Kamata, 2008). In heavily impacted regions, more extreme IPM approaches, such as removal, burning, and fumigation of trees and aerial spray and trunk injection of nematicides and insecticides have been undertaken to control the spread of the disease (Takai et al., 2003; Kwon et al., 2005; Bonifácio et al., 2014). Although, these approaches are effective to some extent, they also negatively impact the forest ecosystems. For example, excessive use of nematicides influences the diversity of other organism because of their nonselective nature, causes environmental hazards, and increases the risk of forest fires (Dou and Yan, 2022). Therefore, it is imperative to establish ecologically friendly methods to reduce the prevalence of PWD.

Biocontrol approaches using biocontrol agents (BCAs) have emerged as sustainable and environmentally friendly viable substitutes for chemical control for the management of PWD and mitigation of PWN impact (Sun et al., 2022; Kamaruzzaman et al., 2024). These strategies provide multiple advantages, such as a smaller ecological footprint, the possibility for long-lasting efficacy, and the ability to target specific pathogens (Dou and Yan, 2022). BCAs (bacteria and fungi) have shown great potential in mitigating PWD through different mechanisms, such as directly killing PWN (Liu et al., 2019b; Yin et al., 2020), producing specific nematicidal and volatile organic compounds (Li et al., 2018; Kang et al., 2021; Kamaruzzaman et al., 2024), and triggering host resistance (Chu et al., 2019; Kim et al., 2019). For example, *Esteya vermicola*, a nematophagous fungus that preys on PWN, has shown promising results in reducing populations of PWN and decreasing the incidence of PWD by killing and parasitizing PWN (Wang et al., 2018; Pires et al., 2022). Similarly, *Beauveria bassiana* and *Metarhizium anisopliae* possess the potential to kill PWN, hence reducing their numbers and halting the spread of PWD (Maehara et al., 2007; Kim et al., 2020b). Members of bacterial genera such as *Bacillus*, *Pseudomonas*, *Curtobacterium*, and *Stenotrophomonas* demonstrated strong nematicidal activities against PWN, mitigate PWD incidence and enhanced the plant growth by producing certain metabolites and volatile organic compounds (VOCs) that are toxic to PWN and by stimulating host immunity (Nascimento et al., 2013; Li et al., 2018; Dou and Yan, 2022). These BCAs can directly be directly applied to diseased trees and provide a viable solution, as they target various stages of the PWN life cycle, hence mitigates PWD in the pine forests. The biocontrol of PWN using fungi as BCAs is more prevalent than that of bacteria, and 100 strains from 51 fungal genera were reported as BCAs compared to bacteria (28 strains of 13 genera) (Dou and Yan, 2022). Thus, the biocontrol of PWD via bacterial BCAs has not yet been fully developed and needs further study.

The plant rhizosphere is recognized as one of the most complex ecosystems on Earth and serves as a hotspot for diverse microbes (Raaijmakers, 2014). The rhizosphere microbiome acts as a first line of defense for plants by establishing a complex community of beneficial microorganisms that can inhibit pathogen invasion (Li et al., 2021). Recently, the host microbiome has gained much attention for improving plant health and disease resistance against pathogens (Trivedi et al., 2020; Ahmed et al., 2022a). Beneficial microorganisms can colonize the host plant, improve nutrient absorption ability, secrete antimicrobial compounds, and stimulate host resistance against many plant pathogens, including PPNs (Ali et al., 2023; Dai et al., 2023). Many previous studies have demonstrated that introducing BCAs significantly mitigates disease incidence by manipulating the host microbiome and increasing the abundance of native microbial communities (Ahmed et al., 2022b; Li et al., 2023b). The microbiome of pine trees consists of diverse of bacterial and fungal communities that play vital roles in disease suppression, nutrient cycling, and plant growth promotion. Previous studies directly explained the biocontrol potential of BCAs (bacteria and fungi) against PWN by extraction of nematicidal compounds, specific secondary metabolites, and VOCs. However, no research has explored the potential of beneficial bacteria as BCAs in mitigating PWD by manipulating the host microbiome. In the present study, we screened a strain of rhizosphere bacteria with strong nematicidal activity against PWN from the rhizosphere soil of a healthy pine (*Pinus massoniana*) plant, and its biocontrol potential was studied against PWD through reshaping the host microbiome via amplicon sequencing. We assume that understanding and harnessing the potential of the host microbiome offers new avenues for developing innovative biocontrol strategies against PWN and improving the overall resilience of pine forests to PWD.

## 2 Materials and methods

### 2.1 Cultivation of pine wood nematode

The pine wood nematode (PWN) *Bursaphelenchus xylophilus* (Steiner and Buhrer, 1934) Nickle 1970 isolate was previously isolated and stored in our laboratory, Guangdong Province Key Laboratory of Microbial Signals and Disease Control, South China Agricultural University, China (Kamaruzzaman et al., 2024). PWN was grown on the mycelium of *Botrytis cinerea* (7-days old culture) cultured at 25°C for one week in the dark on potato dextrose agar. The Baermann funnel method was employed to harvest the PWN from the culture medium (Mannaa and Seo, 2023). The harvested nematodes were thoroughly washed with sterilized distilled water (sdH_2_O) to remove most contaminants from the cuticle and stored at 4°C for future use.

### 2.2 Isolation and purification of bacterial strains

Soil samples were collected from roots of the of healthy *Pinus massoniana* free from PWN infestation, on the campus of South China Agricultural University, Guangzhou, China. Three soil samples were collected from *P. massoniana* and mixed together to create a composite sample. Bacterial strains were isolated from the collected soil sample using a serial dilution method described by Ahmed (Ahmed et al., 2022c). For the isolation of bacterial strains, one gram of soil was thoroughly mixed in 9 mL of sdH_2_O and incubated at 28°C and 180 rpm for 30 minutes. The mixture was subsequently serially diluted (10-folds), and 100 μL of the diluted solution was spread on nutrient agar (NA) media supplemented with 50 mg/L Nystatin (to inhibit fungi growth) with a sterile spreader and incubated at 28°C for two days. On the basis of colony morphology (shape and color), a single colony was picked and streaked on NA medium for purification and 63 rhizobacterial strains were collected. After five rounds of streaking and purification, the purified strains were stored in 50% glycerol stock solution (v/v) at −20°C for future use (strains identification and nematode mortality assays).

### 2.3 Nematicidal potential of isolated bacterial strains against PWN

The isolated bacterial strains were reactivated on NA media at 28°C for 48 hours to determine their nematocidal potential against PWN. The nematicidal activity of the isolated strains was assessed against PWN via the preparation of fermentation filtrates (FFs) according to the methodology of Puttawong et al. (2024). The seeds solutions of the isolated bacterial strains were prepared by adding a single colony from each strain into 50 mL of nutrient broth (NB) and incubating at 28°C for 15 hours. For the preparation of FFs, 100 mL of NB was inoculated with 1% seed solution and incubated on a shaker for 72 hours at 28°C and 180 rpm. The fermentation mixture was subsequently centrifuged at 4°C and 10,000 rpm for 10 minutes, which was repeated twice. The supernatant was collected and subsequently filtered using a 0.22 μm bacterial filter to obtain FFs and then stored at 4°C for later use. We used the immersion method to evaluate the nematicidal activity of FFs of each bacterial strain against PWN (Moo-Koh et al., 2022). A mixture of 10 μL of nematodes suspension (∼100 PWN) and 90 μL of FFs was added to a 48-well cell culture plate (each well) and incubated in the dark at 28°C for 48 hours, whereas sdH_2_O was used as a control. Nematodes morphology was observed every 24 hours using a stereomicroscope (Nikon SMZ 745; Nikon, Tokyo, Japan) to calculate the corrected mortality rate (C-M-R). Nematodes were determined to be dead when their bodies were stiff and unresponsive to stimulation by a dissecting needle (Ali et al., 2023). The C-M-R was calculated via the following formula (Ayaz et al., 2021): CM (%) = (*T*− *CK*)/(1 − *CK*) × 100. Where *T* represents the mortality rate in the treatment group and *CK* represents the mortality rate in the control group. The assay was repeated three times, with minimum three replicates per treatment. From the isolated bacterial strains, strain DP2-30 exhibited strong nematicidal activity against PWN and was selected for subsequent analyses.

### 2.4 Identification and characterization of potent biocontrol strain

The potential biocontrol strain DP2-30 was identified on the basis of morphological (colony shape, color, cell size, and Gram staining) and molecular (PCR amplification and phylogenetic analysis) characteristics. Morphological identification of strain DP2-30 was performed by growing it on NA media at 28°C for 48 hours. Gram staining of strain DP2-30 was performed using a Gram Stain Kit, Solarbio Life Sciences & Technology Co., Ltd. (Beijing, China). The cell morphology and size were observed by Scanning Electron Microscope (SEM; (LEO 1450 VP SEM, Germany) (Taheri et al., 2023). Molecular identification of strain DP2-30 was performed by PCR amplification of 16S *rRNA* gene via primer pair 27F-(5-AGAGTTTGATCCTGGCTCAG-3) and 1492R (5-GGTTACCTTGTTACGACTT-3) (Zhang et al., 2022b). The PCR products were observed using a 1% agarose gel electrophoresis in a gel documentation system (Tanon 2500) and sent to Guangzhou Aiji Biotechnology Co., Ltd. (Guangdong, China) for sequencing. The obtained sequencing results were analyzed by the BLASTN tool in the NCBI database. Mega 11 was used to edit, build, Clustal W, and alignment of sequences, and a phylogenetic tree was constructed with the neighbor-joining method (bootstrap analysis with 1000 replicates) by taking *Bacillus* sp. as an outgroup (Tamura et al., 2021).

### 2.5 Optimizing culture medium conditions to enhance *Luteibacter pinisoli* DP2-30 nematicidal activity against PWN

The effects of different culture medium conditions, such as incubation time (days), temperature (°C), shaking speed (rpm/min), and culture medium pH on the nematicidal activity of the *L. pinisoli* DP2-30 against PWN were assessed to optimize the fermentation conditions to maximize the nematicidal activity (Kamaruzzaman et al., 2024). The seed solution of strain DP2-30 was prepared as described above (Section 2.3), and 1% of the seed solution was used for subsequent analyses. The impact of incubation time on the nematicidal activity of strain DP2-30 was examined by growing strain DP2-30 in 100 mL of NB for 1, 2, 3, 4, 5, 6, and 7 days and the nematicidal activity was checked every 24 hours. To study how the pH of the culture medium influences the nematicidal activity of DP2-30 against PWN, the pH of the culture medium was adjusted to 4.0, 5.0, 6.0, 7.0, 8.0, 9.0, and 10.0. Subsequently, 100 mL of culture mediums (NB) with different pH values were inoculated with strain DP2-30 seed solution and incubated at 180 rpm/min and 28°C for 72 hours. To assess the effect of incubation temperature (°C), 100 mL of culture medium (NB) containing DP2-30 seed solution was cultured at 180 rpm/min and different temperatures 22, 25, 28, 31, 34, and 37°C for 72 hours. Finally, the impact of shaking speed (rpm/min) on the nematicidal activity of strain DP2-30 was determined by culturing 100 mL of NB at 120, 140, 160, 180, 200 and 220 rpm/min and 28°C for 72 hours. The FFs of strain DP2-30 for each assay were collected via centrifugation as mentioned in section 2.3, and their nematicidal activity was assessed as described above (Section 2.3). All the assays were performed three times, with at least three replications per treatment for each assay.

### 2.6 Effect of the *Luteibacter pinisoli* DP2-30 on the success hatching of PWN eggs

The effects of different conditions (FFs, fermentation broth (FB), and bacterial suspension) of strain DP2-30 on PWN eggs hatching were studied as described by Sun et al. (2022). The PWN eggs were collected following the methodology of Liu and colleagues (Liu et al., 2019a) and adjusted to a concentration of 100 eggs per 10 μL via a microscope (Nikon SMZ 745; Nikon, Tokyo, Japan). A 10 μL of egg suspension (contained ~100 PWN eggs) was added to a 48-well cell culture plate containing 90 μL of FFs, FB, and bacterial suspension (OD_600_ = 0.5) and incubated in the dark at 28°C for 36 h, while sdH_2_O was used as a control. The PWN (L2 stage) number was counted under a stereomicroscope (Nikon SMZ 745; Nikon, Tokyo, Japan). The PWN egg hatching rate (HR: %) and relative inhibition rate (IR: %) were calculated using the following formulas: HR (%) = L2 × 100/eggs + L2 and IR (%) = (HR of control – HR of treatment) × 100/HR of control. The experiment was performed three times, with three replication per treatment.

### 2.7 Determining the plant growth-promoting ability of *Luteibacter pinisoli* DP2-30

The growth-promoting potential of strain DP2-30 was assessed on a model plant (tobacco; *Nicotiana benthamiana*). For the growth promotion assay, the FB and FFs of strain DP2-30 were obtained by growing it in NB media at 28°C and 180 rpm/min for 72 hours. Tobacco seeds were first surface sterilized with 10% sodium hypochlorite solution for 5 min, followed by 4 times washing with sdH_2_O (Shoyeb et al., 2020). After surface sterilization, the tobacco seeds (30) were soaked in 2 mL of FFs and FB for 12 hours, while NB and sdH_2_O were used as controls. The seeds (30 seeds per dish, 3 dishes per treatment) were then placed in Petri dishes containing MS media (0.8% agar, 1.5% sucrose, and pH 5.7) and placed in a light incubator at 25°C (12 h light and 12 h dark) under controlled conditions (Sheikh et al., 2023). The assay was performed three times with three dishes per treatment as replicates and each dish contained ten seeds. After 14 days, the seed germination rate, fresh weight, root length, and stem length were recorded and calculated (Sheikh et al., 2023).

### 2.8 *In planta* assay for determining the biocontrol efficacy of *Luteibacter pinisoli* DP2-30 against pine wilt disease and samples collection

The biocontrol potential of strain DP2-30 against PWD was investigated in a greenhouse pot experiment on 2-year-old pine seedlings (*P. massoniana*) artificially inoculated with PWN. Before the experiment, strain DP2-30 was grown in NB medium at 28°C and 180 rpm/min for 72 hours to prepare FB. The experiment was executed following a completely randomized design under three conditions: T1 (inoculation of ∼3000 PWN per mL/plant), T2 (combined application of DP2-30 FB and ∼3000 PWN per mL/plant), and CK (application of sdH_2_O as blank control). We used sdH_2_O as control to establish a baseline for comparison, which allows us to assess the effects of DP2-30 without any confounding variables that might arise from other treatments. Strain DP2-30 FB (30 mL/plant) was inoculated via the root drenching method around the base of pine plant by disturbing the above 1 cm layer, and the PWN suspension was artificially inoculated into the main stem 5 cm below the top with superficial longitudinal incisions in a well-sealed premade parafilm funnel and kept moist (Rodrigues et al., 2021). Disease development was observed at 9 days of post-inoculation with PWN to 45 days with an interval of 9 days. The disease severity index (%) was calculated for each treatment using a 5-point disease grading scale (0: asymptomatic plants, 1: 1−25% wilting of pine needles, 2: 26−50% wilting of pine needles, 3: 51−75% wilting of pine needles, and 4: 76−100% wilting of pine needles or death of plant). The disease severity index (%) and control effect (%) were calculated using formulas as previously described by Kamaruzzaman et al. (2024). The experiment was repeated three times and 8 pine seedlings were inoculated per treatment as replicates for each individual assay. To study, the impact of strain DP2-30 on the host microbiome, particularly in the context of its potential role in managing PWN infestations through modulation of host microbiome. Root and stem samples were collected from the pine seedlings under different treatments from the *in-planta* biocontrol assay. A total of 36 samples (18 roots and 18 stems; 6 samples per treatment) were collected under different treatments (CK, T1, and T2), placed in liquid nitrogen, and stored at −80°C for host-microbiome analysis. The samples collected from each treatment were labelled according to sample type: CK-R, T1-R, and T2-R (roots) and CK-S, T1-S, and T2-S (stem).

### 2.9 DNA extraction, Illumina MiSeq sequencing, and data processing

The total genomic DNA from the plant samples were extracted as previously described in our study (Ahmed et al., 2022a), using a Plant DNA Extraction Kit (Zymo Research, USA). The quality of the extracted DNA was assessed at an OD_A260/A280_ ratio of≥1.8 using an ND2000 nanodrop spectrophotometer (Thermo Scientific, USA). For microbiome analysis, the V5-V7 region of bacterial 16S rRNA gene and fungal ITS1 region were amplified with the primer sets 799F (5-AACMGGATTAGATACCCKG-3)/1193R (5-ACGTCATCCCCACCTTCC-3) (Li et al., 2023b) and ITS1-F_KYO2 (5-TAGAGGAAGTAAAAGTCGTAA-3)/ITS-86R (5-TTCAAAGATTCGATGATTCAC-3) (Toju et al., 2012), respectively. The obtained PCR samples were subjected to paired-end sequencing on an Illumina MiSeq platform at Gene Denovo Biotechnology Co., Ltd. (Guangzhou, China). The raw sequences of bacteria and fungi were analyzed and processed using the UPARSE pipeline according to standard protocols and quality controlled by USEARCH software to generate clean reads (Edgar, 2013). Chimeras were eliminated via UCHIME, and subsequently, the remaining clean sequences were allocated to operational taxonomic units (OTUs) on the basis of a similarity standard of 97% (Edgar et al., 2011). Ultimately, the sequence of each OTU was compared to the RDP database of bacteria and UNITE database of fungi according to the Naïve Bayesian algorithm of the RDP Classifier for taxonomic annotation at a confidence threshold level of 0.8~1 (Wang et al., 2007; Nilsson et al., 2019). The raw data from the greenhouse experiments are available in the NCBI Sequence Read Archive (SRA) under BioProject No. PRJNA1132778 with the sample names of CK-R, T1-R, and T2-R (R; root) and CK-S, T1-S, and T2-S (S; stem).

### 2.10 Statistical and bioinformatics analyses

The alpha diversities of diversities (Shannon, Chao, PD-whole-tree, and Chao-1) bacterial and fungal communities alpha were calculated using QIIME 2 (Bolyen et al., 2019). We used principal coordinate analysis (PCoA) based on the Bray–Curtis distance metric to assess the variations in the structure of the bacterial and fungal community compositions. PERMANOVA with Adonis function was employed to determine the overall dissimilarity in the structure of the microbial communities using “vegan” package in R (version 4.0.1) (Oksanen et al., 2013). The relative abundance bar, box, and bubble plots for the bacterial and fungal communities were generated using the “ggplot” package in R (version 4.0.1). The intra-kingdom microbial network analysis was performed at the OTU level by excluding those with a relative abundance < 0.01, *p* < 0.05, and Pearson correlation coefficient > 0.7 using R sparcc package and visualized with Gephi 0.9.2 (Zhang et al., 2024). We employed one-way ANOVA in SPSS v20.0 (SPSS Inc. USA) to evaluate the impact of different treatments on the disease severity index, nematicidal activity, alpha diversity, and microbial community composition and were considered significant if *p* < 0.05 according to Tukey-HSD and Duncan’s multiple range tests. The figures were edited, merged, and combined using Adobe Illustrator 2022.

## 3 Results

### 3.1 Isolation and characterization of *Luteibacter pinisoli* DP2-30

**Figure 1:**
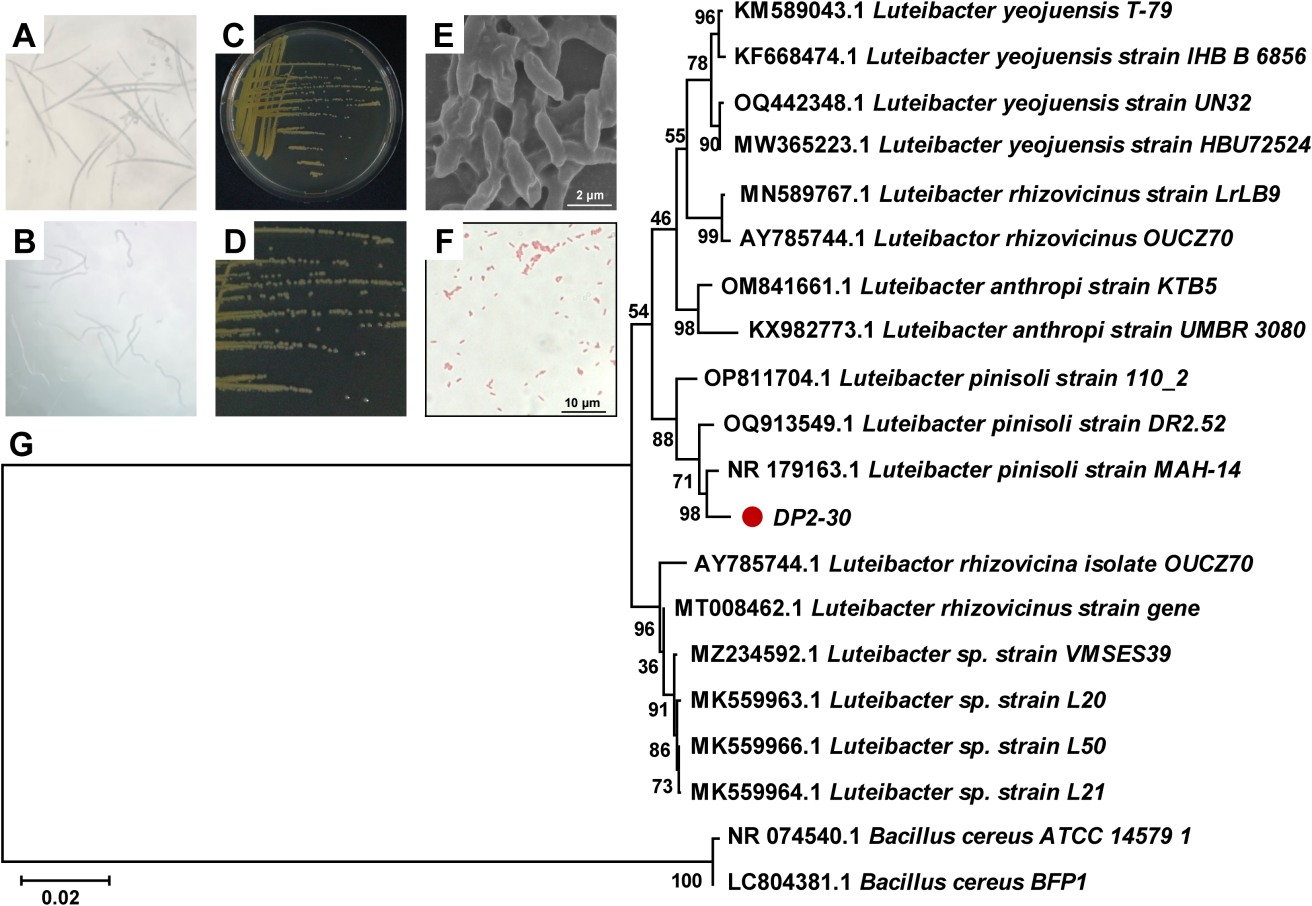
Nematicidal activity, morphological, and molecular characterization of *Luteibacter pinisoli* DP2-30. Nematicidal activity of strain DP2-30 against PWN **(A)**, control **(B)**, colony morphology on NA medium **(C, D)**, scanning electron microscope image of bacterial cells **(E)**, Gram staining **(F)**, and phylogenetic tree based on 16S rRNA sequence illustrating the evolutionary position of strain DP2-30 via neighbor-joining method with a bootstrap value of 1000.

A total of 63 bacterial strains were preliminarily isolated and purified from the rhizosphere soil of healthy *Pinus massoniana* plants. The nematicidal activity of isolated bacterial strains was investigated against PWN using fermentation filtrates (FFs) by the immersion method, among which 10 bacterial strains showed various degree of nematicidal activity (20.70% to 92.08%) against PWN after 48 h of exposure. The strain DP2-30 displayed the highest nematicidal activity with a corrected mortality rate (C-M-R) of 92.08% against PWN (**Figures 1A, B**), followed by strains DP1-4 and DP2-15 with C-M-R of 75.01% and 73.94% (moderate nematicidal activity), respectively. Several strains, including DP2-13, DP2-27, DP1-2, DP1-18, Dp2-2, DP2-24, and DP2-41 exhibited low nematicidal activity against PWN, with C-M-R values less than 40% (**Table 1**). In light of these findings, we therefore, chose DP2-30 as a possible biocontrol strain for further investigation. Morphological and biochemical analyses were performed for strain DP2-30. On NA media strain DP2-30 produced smooth, opaque, and round to convex yellow color colonies (**Figures 1C, D**). Scanning electron microscopy revealed that the bacterial cells were rod-shaped with 1.19 ± 0.20 μm in length and 0.37 ± 0.03 μm in diameter, and Gram staining was negative (**Figures 1E, F**). Molecular identification of strain DP2-30 was performed via PCR amplification of the 16S rDNA gene using primer set 27F/1492R. A 938 bp PCR product was obtained, and NCBI BLASTN analysis showed 98.80% similarity with *Luteibacter pinisoli* MAH-14 (NR_179163.1). Further phylogenetic tree analysis via neighbor-joining method revealed that strain DP2-30 was in the same clade as *L. pinisoli* and was different from other *Luteibacter* species (**Figure 1G**). Thus, on the basis of morphological, biochemical, and molecular analyses, the isolated strain DP2-30 was determined to be *L. pinisoli* and was named as *L. pinisoli* P2-30.

**Table 1:** Nematicidal activity of fermentation filtrates of isolated bacterial strains on pine wood nematode in a co-culture assay.

Strains	48 h mortality rate (%)	48 h corrected mortality rate (%)
DP1-2	38.01 ± 2.16 b	35.70 ± 2.24 b
DP 1-4	75.90 ± 2.52 ab	75.01 ± 2.61 ab
DP1-18	70.33 ± 2.93 b	69.22 ± 2.93 b
DP 2-2	35.19 ± 5.96 b	32.78 ± 6.18 b
DP2-13	57.72 ± 3.81 ab	56.14 ± 3.96 ab
DP2-15	74.87 ± 4.61 ab	73.94 ± 4.78 ab
DP2-24	34.12 ± 2.95 b	31.67 ± 3.06 b
DP2-27	56.21 ± 1.23 b	54.58 ± 1.28 b
DP2-30	92.36 ± 1.26 a	92.08 ± 1.31 a
DP2-41	23.55 ± 1.95 b	20.70 ± 2.02 b
CK-NB	12.66 ± 3.06 bc	—–
CK-sdH_2_O	9.14 ± 1.27 c	—–

Different letters within a column indicate significant differences among bacterial strains according to the Duncans multiple range test at *p* < 0.5.

### 3.2 Fermentation conditions for maximal nematicidal activity of *Luteibacter pinisoli* DP2-30

**Figure 2:**
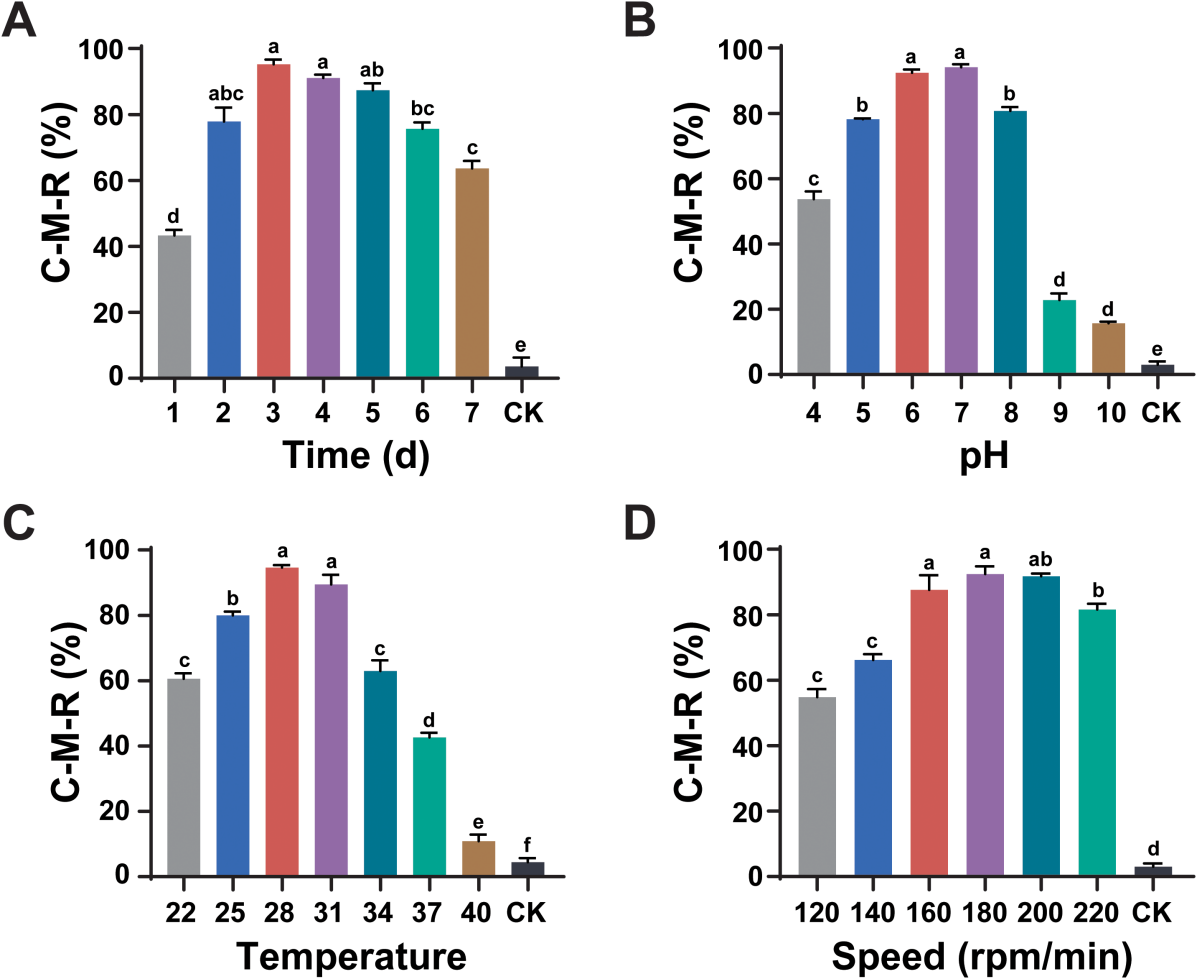
Optimization of fermentation conditions for maximal nematicidal activity of *Luteibacter pinisoli* DP2-30. Effects of different fermentation conditions, including incubation time **(A)**, pH **(B)**, temperature °C **(C)**, and rotation speed (rpm/min) **(D)**, on the nematicidal activity of *L. pinisoli* DP2-30 against PWN. Different letters on the error bars (± SD of three biological replicates) indicate significant differences among treatments according to Duncan’s test at *p* < 0.05.

The effects of various fermentation conditions, including culture medium time, pH, temperature, and shaker speed on the nematicidal activity of *L. pinisoli* DP2-30 were assessed against pine wood nematode (PWN) using fermentation filtrates (FFs). Results revealed that the nematicidal activity of strain DP2-30 FFs first increased with the increasing in incubation time, temperature, speed, and fermentation medium pH and then gradually decreased (**Figures 2A–D**). The nematocidal activity of strain DP2-30 FFs was the highest against PWN at 3 d of incubation with a C-M-R of 95.21% (**Figure 2A**). At pH 7, the nematicidal activity of strain DP2-30 FFs was maximum, with a C-M-R of 94.10% (**Figure 2B**). When the incubation temperature was 28°C, strain DP2-30 FFs presented the highest killing rate against PWN with a C-M-R of 94.60% (**Figure 2C**). The FFs of strain DP2-30 exhibited strong nematicidal activity against PWN when cultured at 180 rpm/min and 200 rpm/min, having a C-M-R values of 92.43% and 91.71%, respectively (**Figure 2D**). On the basis of these findings, the optimal fermentation conditions for maximizing the nematicidal activity of *L. pinisoli* DP2-30 against PWN appear to be 3 days of incubation at 28°C, pH 7, and a shaking speed of 180 rpm/min.

### 3.3 *Luteibacter pinisoli* DP2-30 fermentation broth inhibits PWN egg-hatching

The effects of strain DP2-30 fermentation broth (FB), FFs, and bacterial suspension on PWN egg hatching were calculated after 36 hours of incubation at 28°C. The FB and FFs significantly reduced PWN egg hatching compared to the control group. The hatchability rates were recorded at 41.17% and 46.33% for the FB and FFS, respectively, which were substantially lower than the control. In contrast, the bacterial suspension of strain DP2-30 itself had only a slight inhibitory effect on egg hatching, with a hatchability rate slightly lower than that of the control group. The FB and FFs demonstrated a relatively high inhibition rates of 49.38% and 43.055%, respectively, on PWN egg hatching compared to the control (**Table 2**). These results indicate that the metabolites produced during fermentation by strain DP2-30, rather than the bacteria themselves, are responsible for the significant inhibition of PWN egg hatching.

**Table 2:** Impact of *Luteibacter pinisoli* DP2-30 on pine wood nematode egg hatching.

Treatments	Egg hatching rate (%)	Relative inhibition rate (%)
Fermentation broth	41.17 ± 1.03b	49.38 ± 1.54b
Fermentation filtrate	46.33 ± 1.71b	43.05 ± 1.54b
Bacterial suspension	77.25 ± 0.94a	5.01 ± 1.81a
Sterile distilled water (CK)	82.00 ± 0.94a	—–

Data are present as mean ± SD of three biological replicates. Different letters within a column indicated significant differences among treatments according to Duncan’s test at *p* < 0.05.

### 3.4 Growth-promoting effects of *Luteibacter pinisoli* DP2-30 on tobacco as model plant

The effects of strain DP2-30 FB and FFs were evaluated on tobacco seed germination and seedling growth (**Supplementary Table S1**; **Supplementary Figure S1**). After the tobacco seeds were soaked in strain DP2-30 FB, FFs, nutrient broth (NB medium) and sdH_2_O for 12 h, they germinated normally and no significant effects were observed among treatments. The germination rate reached more than 90.00% in all treatments, and no significant difference was observed among treatments, indicating that strain DP2-30 did not affect the tobacco seed germination rate. After 14 days of tobacco seed growth in the dish, the tobacco seeds in the treatment group had stronger buds, thicker green leaves, longer roots, and greater fresh weight than the control group (**Supplementary Figure S1**). The root length of tobacco seedlings treated with FB was significantly increased by 75.05% and 74.30% compared with control groups, while the fresh weight was increased by 58.6% and 61.4%, respectively. Similarly, the root length of tobacco seedlings treated with FFs significantly increased by 60.4% and 59.8% compared with control groups, whereas the fresh weight increased by 49.1% and 51.8%, respectively (**Supplementary Table S1**). The results showed that strain DP2-30 significantly promote the growth of the tobacco seedlings, and could be used as a potential plant growth promoting agent.

### 3.5 *Luteibacter pinisoli* DP2-30 fermentation broth reduces pine wilt disease incidence in pine seedlings

**Figure 3:**
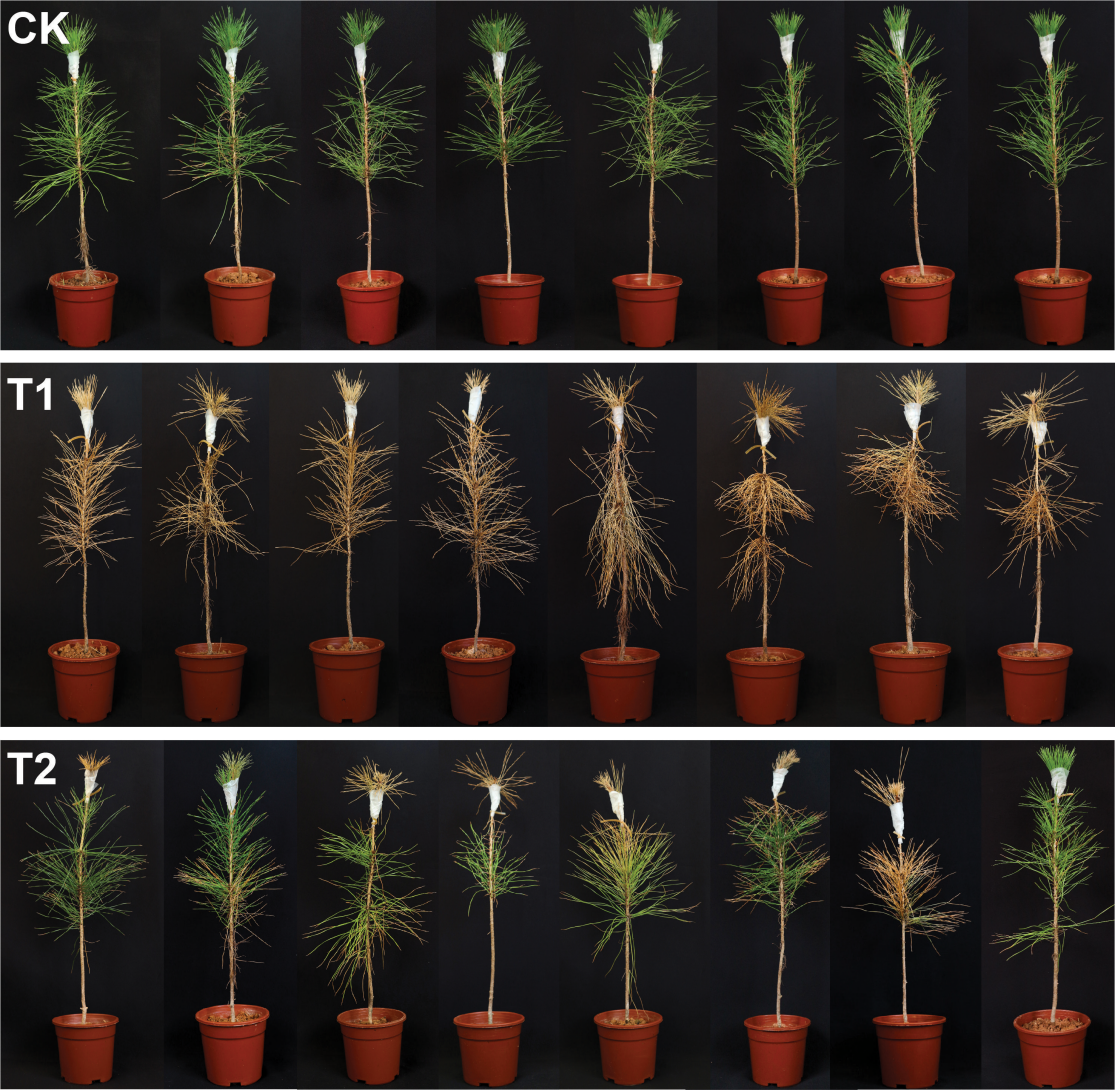
Biocontrol potential of *Luteibacter pinisoli* DP2-30 fermentation broth against pine wilt disease in a greenhouse assay. Here: CK; application of sdH2O, T1; application of PWN, and T2; combined application of *L. pinisoli* DP2-30 fermentation broth + PWN. Different letters on the error bars represent significant differences among treatments according to Duncan’s test at *p* < 0.05. Data related to the disease severity index (%) and control effect (%) are presented in **Table 3**.

The biocontrol efficacy of *L. pinisoli* DP2-30 was assessed in mitigating PWD in pine seedlings treated with PWN and strain DP2-30 FB. The disease severity index (%) was calculated from the 9^th^ day after inoculation with PWN till the 45^th^ days, with the interval of 9 days, whereas the control effect (%) was calculated at the end of the experiment (**Table 3** and **Figure 3**). The results indicated that symptom of PWD (fade, yellowing, and wilting of needles) were first observed on the 9^th^ day post-inoculation with PWN in treatment T1 (pine seedlings solely inoculated with PWN). In contrast, the application of DP2-30 FB (T2; combined treatment with DP2-30 FB and PWN) resulted in delayed symptom development in pine seedlings, and PWD symptoms were first observed on the 27^th^ after inoculation with PWN. The disease incidence of PWD gradually increased with the increasing time in treatments T1 and T2, whereas the control plants (CK) treated with sdH2O exhibited normal growth, were healthy, and remained lush green (**Figure 3**). At 45 days after inoculation with PWN, the highest disease severity index of 100% was observed in treatment T1. However, pine seedlings treated with DP2-30 FB + PWN (T2) displayed a minimum disease severity index of 37.5% and maximum control effect of 62.50% (**Table 3**). These results showed that the application of DP2-30 FB (T2) significantly delayed the occurrence of PWD in pine seedlings.

**Table 3:** Control effect of *Luteibacter pinisoli* DP2-30 fermentation broth on incidence of pine wilt disease.

Treatments	Disease severity index (%)					Control effect %
	9d	18d	27d	36d	45d	
T1	15.63 ± 1.23a	34.38 ± 2.52a	59.38 ± 3.51a	84.37 ± 2.92a	100.00 ± 0.00a	—–
T2	0.00 ± 0.00b	0.00 ± 0.00b	12.50 ± 1.24b	21.86 ± 1.94b	37.50 ± 2.24b	62.50 ± 1.79a
CK	0.00 ± 0.00b	0.00 ± 0.00b	0.00 ± 0.00c	0.00 ± 0.00c	0.00 ± 0.00c	—–

Here: T1; sdH_2_O + PWN, T2; *L. pinisoli* DP2-30 fermentation broth + PWN, and CK; sdH_2_O. Data are present as mean ± SD of three biological replicates. Lowercase letters in each column show significant differences among treatments according to Duncans’s test at *p* < 0.05.

### 3.6 Pine plant microbial community assembly, diversity, and structure analyses

**Figure 4:**
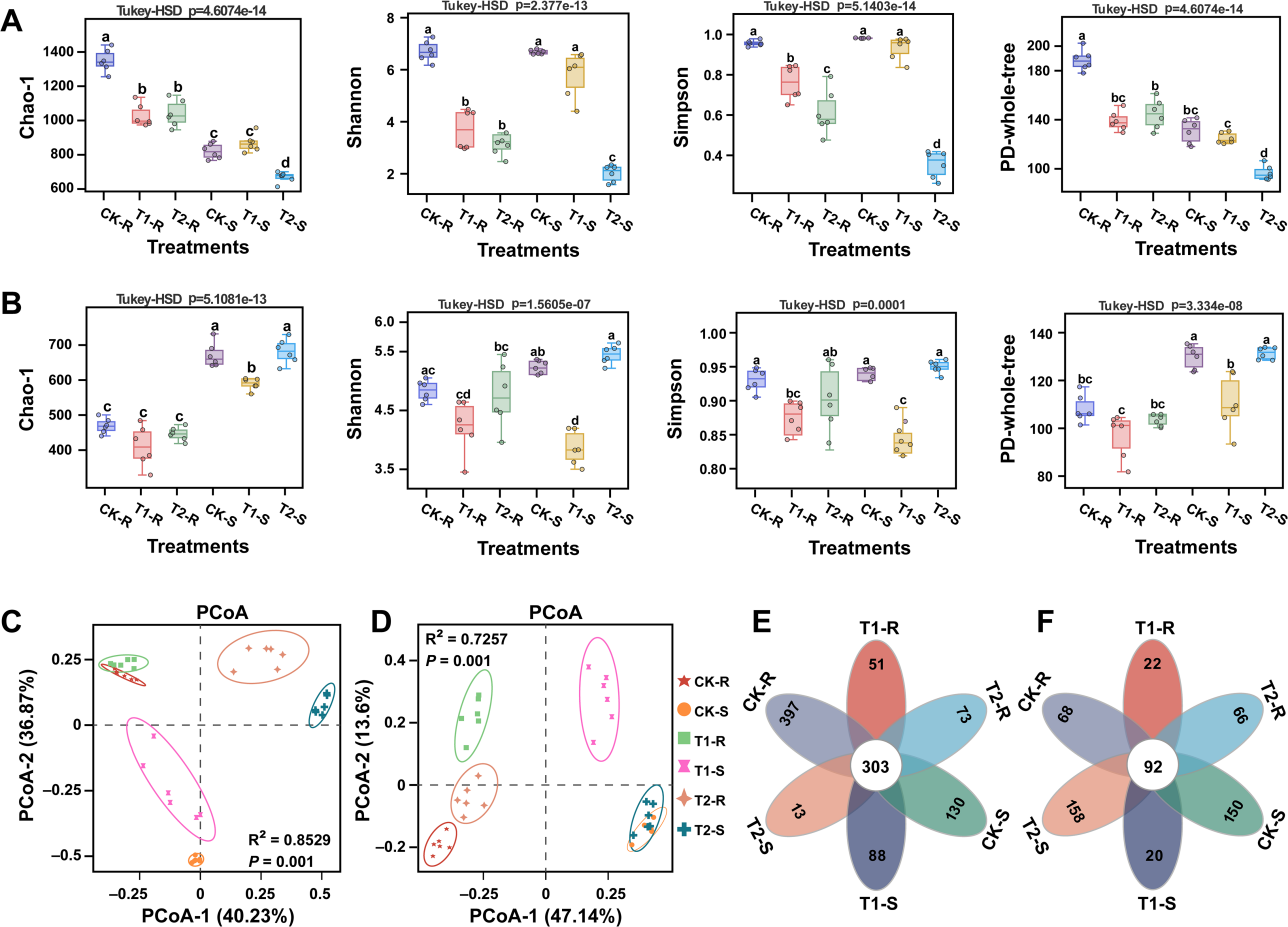
Diversity and structure of root and stem-associated microbial communities under different treatments. Alpha diversity metrics of bacterial **(A)** and fungal **(B)** communities under different treatments. Principal coordinate analysis (PCoA) based on Bray-Curtis dissimilarity metrics and permutational multivariate analysis of variance (PERMANOVA) showing the distance in the bacterial **(C)** and fungal **(D)** communities between the treatments. The flower Venn diagram displays the common and unique bacterial **(E)** and fungal **(F)** OTUs under different treatments. Here, CK; application of water as control, T1; application of PWNs, and T2; combined application of PWNs + *L. pinisoli* DP2-30. R, root; S, stem. Different letters on the error bars indicate significant differences among treatments according to the Tukey-HSD test *p* < 0.05.

Thirty-six samples (18 roots and 18 stems) of pine plants from different treatments (CK, T1, and T2) were subjected on the Illumina platform for analysis of the host endophytic microbiome (bacteria and fungi). The 16S rRNA and ITS amplicon sequencing of V5-V7 and ITS1 variable regions of bacteria and fungi resulted in a total of 4.547 and 4.523 million raw reads, respectively. After quality control and chimera removal, 3.285 and 4.228 million effective tags of bacteria and fungi were obtained, with an average of 0.091 and 0.117 million effective tags per sample of bacteria and fungi, respectively. These effective tags were then clustered into 29909 bacterial and 16952 fungal OTUs for taxonomic annotation, with an average of 830 bacterial and 470 fungal OTUs per sample (**Supplementary Tables S2, S3**). The obtained OTUs were used to calculate the alpha diversity indices (Chao-1, Shannon, Simpson, and PD-whole-tree indices) and the beta diversity (changes in the structure) of the bacterial and fungal communities in different samples. A significant difference was observed in the alpha diversity indices of bacterial and fungal communities among the samples under different treatments (**Figures 4A, B**; Tukey-HSD, *p* < 0.05). The values of alpha diversity indices for bacterial communities under treatment T2 (T2-S) were significantly decreased as compared to other treatments (**Figure 4A**; Tukey-HSD, *p* < 0.05), while for fungal community values of alpha diversity indices were increased dramatically under treatment T2 (T2-S) than other treatments (**Figure 4B**; Tukey-HSD, *p* < 0.05). PCoA based on Bray–Curti’s dissimilarity matrix and PERMANOVA (Adonis) demonstrated a significant difference in the structure of bacterial and fungal communities under different treatments. The first two components of PCoA explained 77.10% (PERMANOVA, *p* = 0.001 and R^2^ = 0.8259) and 60.74% (PERMANOVA, *p* = 0.001 and R^2^ = 0.7257) of the variation in the structure of the bacterial and fungal communities, respectively under different treatments (**Figures 4C, D**; **Supplementary Table S4**). First, we generated Upset plots to show the shared and unique bacterial and fungal OTUs among samples (roots and stems) under different treatments (**Supplementary Figures S2A–C**). The flower Venn diagram also revealed 303 bacterial and 92 fungal shared OTUs among the samples (roots and stems) under the different treatments. Further OTUs analysis revealed that 397, 51, 73, 130, 88, and 13 bacterial and 68, 22, 66, 150, 20, and 158 fungal OTUs were presented as unique OTUs in CK-R, T1-R, T2-R, CK-S, T1-S, and T2-S, respectively (**Figures 4E, F**). These results suggested that the variations in the diversity and structure of the bacterial and fungal communities might be due to these common and shared OTUs.

### 3.7 Variations in the microbial community composition under different treatments

**Figure 5:**
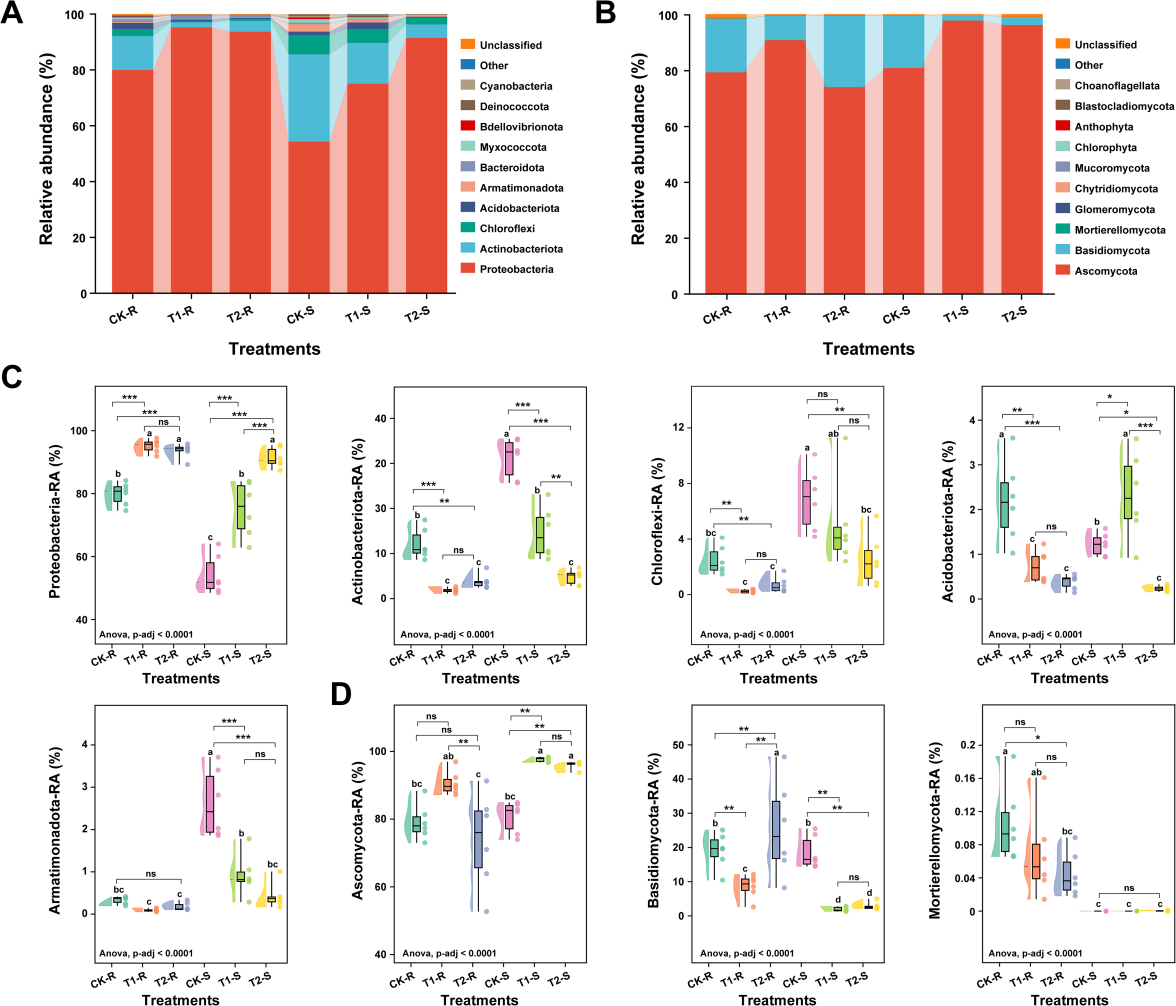
Relative abundance of the most abundant bacterial and fungal communities at the phylum level. Stacked bar graphs showing the variations in the relative abundance (average, n=6) of the top 10 bacterial **(A)** and fungal **(B)** phyla under different treatments. Box plots displaying the significant differences in the relative abundance most dominant bacterial **(C)** and fungal **(D)** under different treatments. Here, CK; application of water as control, T1; application of PWNs, and T2; combined application of PWNs + *L. pinisoli* DP2-30. R, root; S, stem. Different letters on the box plots show significant differences among treatments according to the Tukey-HSD test at *p* < 0.05. According to Tukey-HSD, asterisks indicate statistically significant differences at *p < 0.05, **p < 0.001, and ***p < 0.001. ns, non significant.

**Figure 6:**
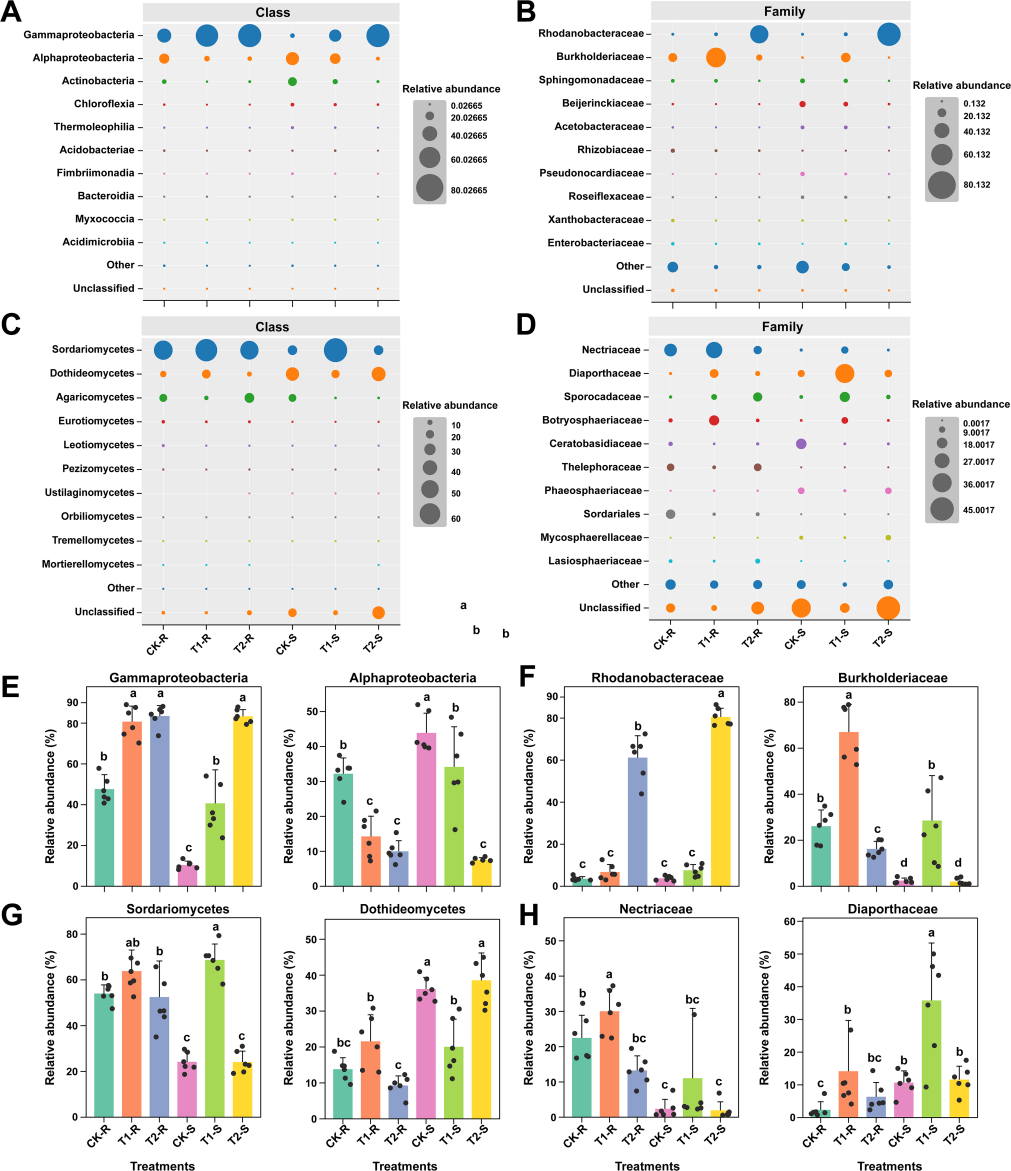
Variations in the relative abundance of microbial communities at the class and family levels under different treatments. Bubble plots illustrating the relative abundance (%) of the most abundant bacterial classes and families **(A, B)** and fungal classes and families **(C, D)** across the treatments. Bar plots showing the significant differences in the relative abundance of dominant bacterial classes and families **(E, F)** and fungal classes and families **(G, H)**. Here, CK; application of water as control, T1; application of PWNs, and T2; combined application of PWNs + *L. pinisoli* DP2-30. R, root; S, stem. Different letters on the error bars indicate the significant differences among treatments according to the Tukey-HSD test at *p* < 0.05.

Analysis of the microbial community composition across different treatments revealed significant variations in the relative abundances of major bacterial and fungal communities at the phylum, class, and family levels in the samples (root and stem) (ANOVA, *p*-adjusted < 0.0001; **Figures 5**, **6**). We detected the top 10 bacterial and fungal phyla in the samples (root and stem) under different treatments and their average relative abundance (RA) in each sample is shown by taxon bar plots (**Figures 5A, B**). Bacterial phylum, such as Proteobacteria, Actinobacteriota, Chloroflexi, and Acidobacteriota and fungal phylum, including Ascomycota and Basidiomycota were the dominant microbial phyla in all the samples with an average RA > 1% across the treatments. Phylum Proteobacteria and Actinobacteriota accounted for 81.63% and 11.45% of the host total bacteriome, respectively (**Figure 5A**), and the phylum Ascomycota and Basidiomycota described 86.38% and 12.82% of host total fungal communities, respectively (**Figure 5B**). Further box plots were constructed to observe the overall variations in microbial community compositions of the most abundant bacterial and fungal phyla among the treatments. Phylum Proteobacteria was present in significantly high RA in T1-R, T2-R, and T2-S compared to CK-R, CK-S, and T1-S (Tukey-HSD, *p* < 0.05). The RA of phyla Actinobacteriota, Chloroflexi, and Armatimonadota was significantly higher in CK-S, and Acidobacteriota was the dominated bacterial phyla in CK-R and T1-S than other treatments (Tukey-HSD, *p* < 0.05; **Figure 5C**). In the fungal communities, the RA of phylum Ascomycota varied significantly among treatments (*p* < 0.05) and was present in significantly high RA in T1-R, T1-S, and T2-S samples compared to others CK-R, T2-R, and CK-S. The RA of phylum Basidiomycota was substantially higher in T2-R than others, and phylum Mortierellomycota was present in significantly higher RA in CK-R than others (Tukey-HSD, *p* < 0.05; **Figure 5D**).

At the class and family levels, the taxonomic distribution patterns of bacterial and fungal communities became more apparent (Tukey-HSD, *p* < 0.05; **Figure 6**). The RA of the top 10 most abundant bacterial and fungal communities at class and family levels are shown by bubble plots (**Figures 6A–D**). Among the bacterial communities, Gammaproteobacteria and Alphaproteobacteria were the highly abundant classes, and Rhodanobacteraceae and Burkholderiaceae were the most abundant families in all samples across the treatments. The class Gammaproteobacteria was present in significantly high RA in T1-R, T2-R, and T2-S as compared to other samples and class Alphaproteobacteria was significantly dominated in CK-S than in the other samples (Tukey-HSD, *p* < 0.05; **Figure 6E**). The RA of the family Rhodanobacteraceae increased considerably in T2-S followed by T2-R compared with CK-R, CK-S, T1-R, and T1-S, and the family Burkholderiaceae was present at a high RA in T1-R than in the other samples (Tukey-HSD, *p* < 0.05; **Figure 6F**). Among the fungal community’s classes, Sordariomycetes and Dothideomycetes and the families Nectriaceae and Diaporthaceae were present at high RA in all samples among the treatments. The class Sordariomycetes had significantly higher RA in T1-S than in the other samples, and Sordariomycetes exhibited in significantly greater RA in CK-S and T2-S than in the other samples under different treatments. The family Nectriaceae was more dominant in the T1-R sample than other samples, and the family Diaporthaceae was more abundant in the T1-S sample than in the other samples across the treatments (Tukey-HSD, *p* < 0.05; **Figures 6G, H**). Analysis of the bacterial community composition at the family level revealed that the application of biocontrol agent *L. pinisoli* DP2-30 significantly increased the RA of the family Rhodanobacteraceae in the roots and stems (T2-R and T2-S) of pine seedlings, as *L. pinisoli* belongs to the family Rhodanobacteraceae.

### 3.8 Intra-kingdom microbial co‐occurrence network analysis of pine plants under different treatments

**Figure 7:**
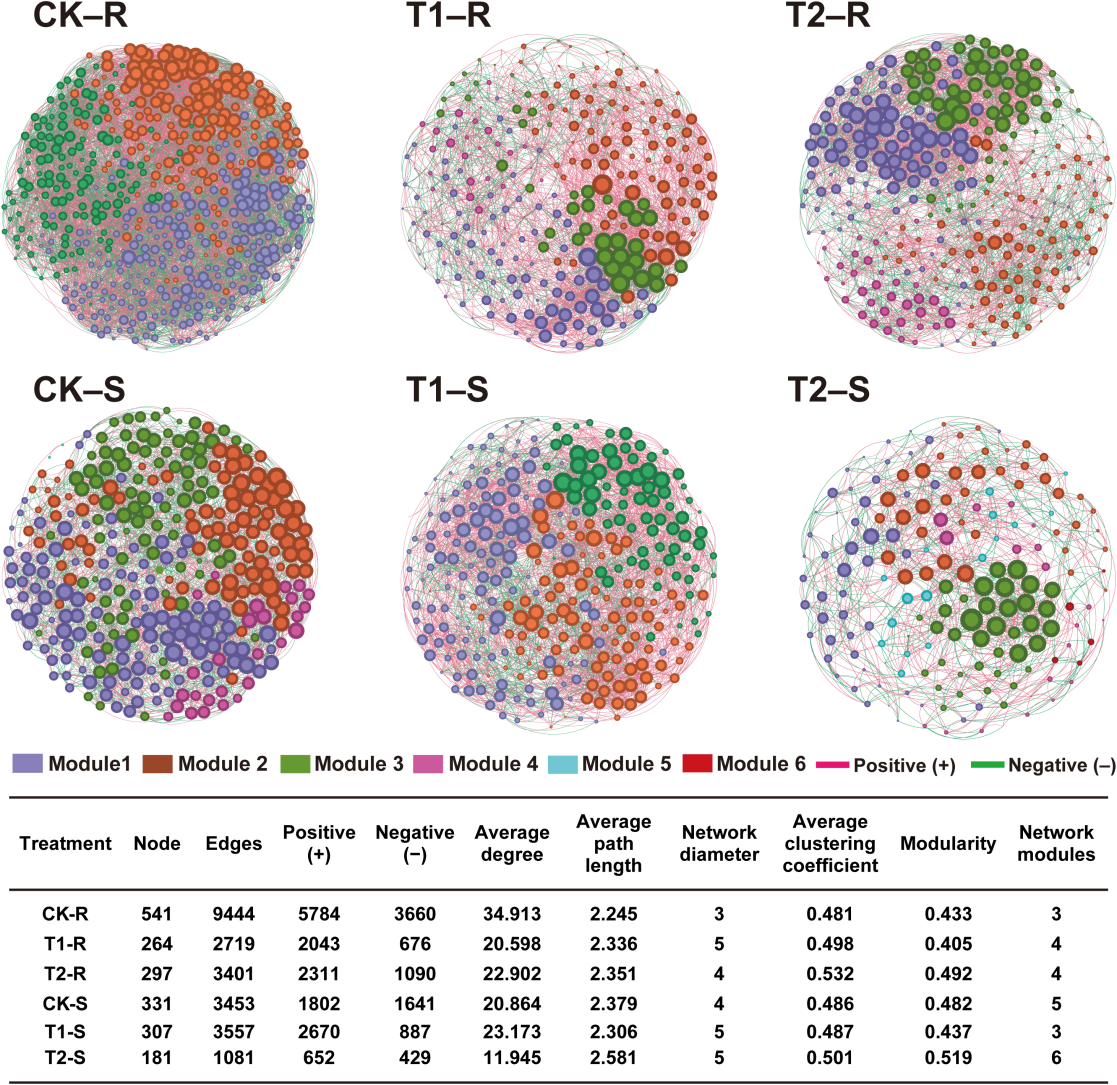
Intra-kingdom (bacteria-bacteria) co‐occurrence network analysis under different treatments. The topological characteristics of the co-occurrence network within each compartment niche are listed in the table. Red and green lines in the networks represent the significant positive and negative relationships among bacterial communities (Spearman’s correlation, *p* < 0.05). Network analysis was performed at the OTU level by excluding the relative abundance < 0.01 (*p* < 0.05 and correlation coefficient > 0.7). Here, CK; application of water as a control, T1; application of PWNs, and T2; combined application of PWNs + *L. pinisoli* DP2-30. R, root; S, stem.

**Figure 8:**
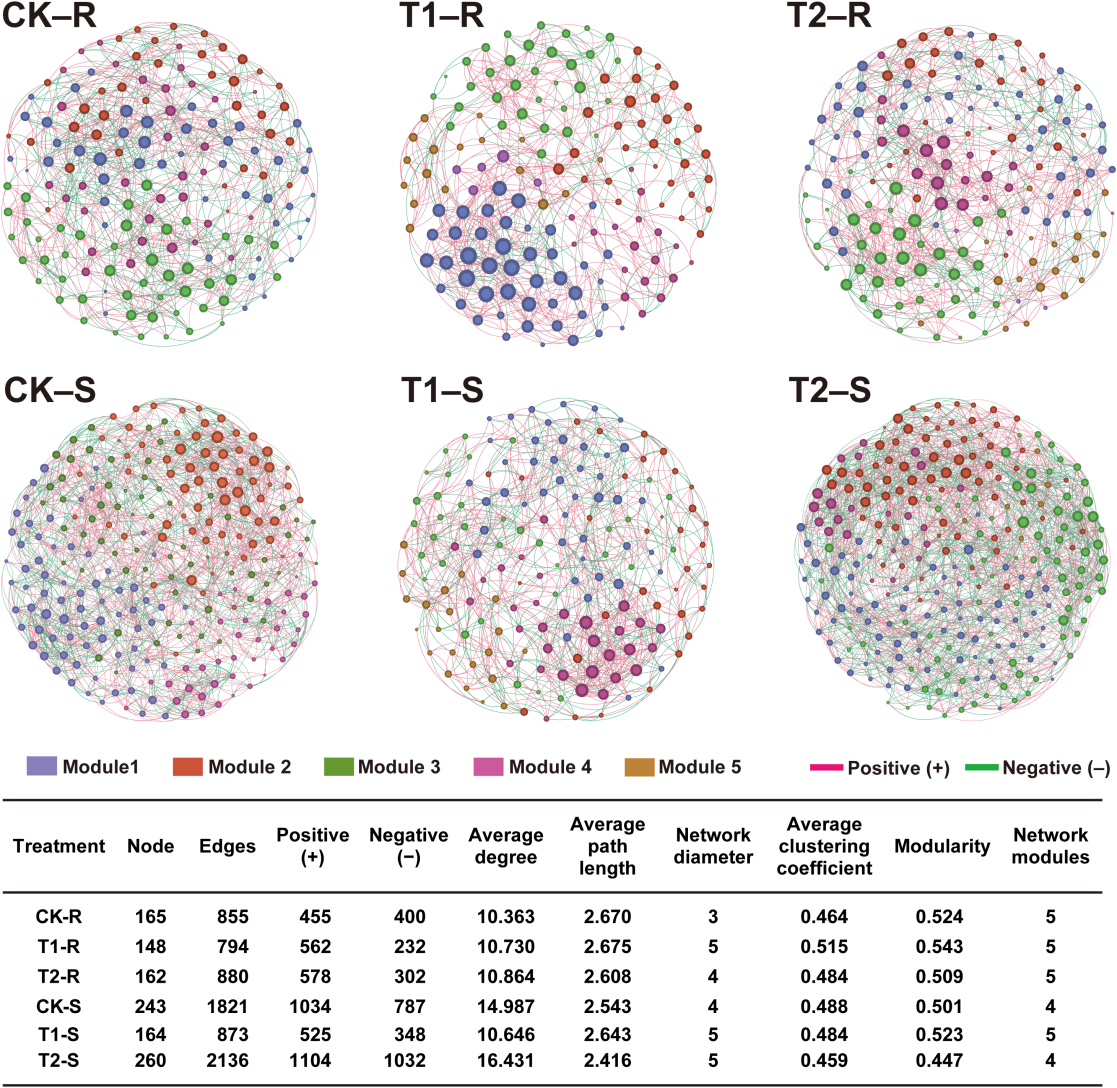
Intra-kingdom (fungi-fungi) co‐occurrence network analysis under different treatments. The topological characteristics of the co-occurrence network within each compartment niche are listed in the table. Red and green lines in the networks represent the significant positive and negative relationships among bacterial communities (Spearman’s correlation, *p* < 0.05). Network analysis was performed at the OTU level by excluding the relative abundance < 0.01 (*p* < 0.05 and correlation coefficient > 0.7). Here, CK; application of water as control, T1; application of PWNs, and T2; combined application of PWNs + *L. pinisoli* DP2-30. R, root; S, stem.

To further explore the impact of different treatments on the host microbiome, we assessed the intra-kingdom co‐occurrence patterns of the bacterial and fungal communities in the different components (root and stem) of the host plants (**Figures 7**, **8**). For bacterial communities, the control group (CK) exhibited a complex co‐occurrence network; however, the complexity of the bacterial co‐occurrence network was reduced significantly from the root to stem in CK and T2 than in T1, in which the network complexity increased (**Figure 7**). For fungal communities treatment T2 showed a more complex co‐occurrence network as compared to CK and T1, and network complexity increased from the root to stem (**Figure 8**). The modularity of all networks was calculated to be >0.4, which suggests a modular network structure of bacterial and fungal communities (**Figures 7**, **8**). The bacterial co‐occurrence network of CK-R and T1-S was divided into 3 modules, which showed a more complex network with a maximum number of nodes (CK-R; 541, T1-S; 307), edges (CK-R; 9444, T1-S; 3557), and average degree (CK-R; 34.913, T1-S; 23.173). In contrast, the networks of T1-R (nodes; 264, edges; 2719, and average degree; 20.598), T2-R (nodes; 297, edges; 3401, and average degree; 22.902), CK-S (nodes; 331, edges; 3453, and average degree; 20.864), and T2-S (nodes; 181, edges; 1081, and average degree; 11.945) were divided into 4, 4, 5, and 6 modules, respectively which demonstrated a decrease in the network complexity. The number of nodes decreased in CK and T2 from the root to stem but increased in T1 from the root to stem. A higher number of positive edges were observed in T1-S (2670) than in CK-S (1802) and T2-S (652), which increased in the order T1-S > CK-S > T2-S (**Figure 7**). In contrast, the complexity of the fungal co‐occurrence network was increased from root to stem under different treatments. A highly complex microbial co-occurrence network was observed for fungal communities in T2-S S (nodes; 260, edges; 2163, average degree; 16.431, and module; 4) and CK-S (nodes; 243, edges; 1821, average degree; 14.987, and module; 4). In comparison, the networks of CK-R (nodes; 165, edges; 855, average degree; 10.363), T1-R (nodes; 148, edges; 794, average degree; 10.730), T2-R (nodes; 162, edges; 880, average degree; 10.864), and T1-S (nodes; 164, edges; 873, average degree; 10.646) were divided into 5 modules, which showed less complexity in co-occurrence network. In the stem, the number of positive edges decreased from T2-S (1104) to CK-S (1034) to T1-R (525), while in root, the number of positive edges decreased in order CK-R (455) < T1-R (562) < T2-R (578) (**Figure 8**). These results suggest that the application of *L. pinisoli* DP2-30 significantly reduced the positive correlation between the PWN and bacterial communities, which resulted in low disease development. The decrease in complexity of the T2 bacterial co‐occurrence network might be due to the strong colonization *L. pinisoli* DP2-30 ability in the host plant, as it significantly enhances the RA of Rhodanobacteraceae family in the host plant. Moreover, *L. pinisoli* DP2-30 make the fungal network more complex by recruiting more fungal communities that plays an important role in disease suppression.

## 4 Discussion

Pine wilt disease (PWD) caused by *B. xylophilus* is a severe threat to pine forests around the globe, affecting forest ecology, natural biodiversity and habitats, climate, and timber production (Pires et al., 2022). Thus, it is necessary to manage the spread of this devastating disease through the application of environmentally friendly management measures to save the natural ecosystem and biodiversity. Pine trees provide habitats for diverse microbial communities, which are crucial for plant health and can suppress by combating pests, thus showing great potential for use as biocontrol agents (Liu et al., 2019b; Kamaruzzaman et al., 2024). This study aimed to explore the potential of *Luteibacter pinisoli* DP2-30 in mitigating PWD via direct antagonism and manipulation of the host microbiome.

Sixty-three bacterial strains were isolated from the rhizosphere soil of healthy *P. massoniana*. Among these strains, DP2-30 showed the highest nematicidal activity against pine wood nematode (PWN), with a C-M-R of 92.08% after 48 hours of treatment. The strain DP2-30 has been identified as *L. pinisoli* through morphological, biochemical, and molecular studies and is identical to a strain previously isolated from the rhizosphere of *P. koraiensis* (Akter and Huq, 2018). The significant nematicidal activity observed in this study is consistent with earlier research that has shown the potential of bacterial BCAs in effectively managing PPNs in different plants (Liu et al., 2019b; Sun et al., 2022; Ali et al., 2023). To further investigate the biocontrol ability of *L. pinisoli* DP2-30 against PWN, we modified the fermentation conditions to maximize the nematicidal activity of strain DP2-30 fermentation filtrates (FFs) against PWN. The results of optimizing fermentation conditions for *L. pinisoli* DP2-30 showed that the highest level of nematicidal activity (>95%) of FFs was obtained after 3 days of incubation at a temperature of 28°C, a pH of 7, and a shaking speed of 180 rpm. These findings emphasize the importance of improving fermentation conditions to increase the biocontrol efficiency of BCAs against phytopathogens. Many other studies have highlighted the impact of these environmental factors on enhancing the biocontrol potential of BCAs (Spadaro et al., 2010; Liu et al., 2017; Kamaruzzaman et al., 2024).

The application of *L. pinisoli* DP2-30 fermentation broth (FB) and FFs resulted in 49.38% and 43.05% decreases in the hatching of PWN eggs, respectively, compared to the bacterium itself (5.01%) and control. We assumed that this inhibitory effect was mainly due to the metabolites produced during fermentation rather than the bacteria (DP2-30). The substantial suppression of PWN egg hatching by fermentation metabolites indicates that secondary metabolites play an important role in inhibiting nematode growth; a similar phenomenon has also been reported in other rhizobacteria, such as *Lysinimonas* sp. M4. The nematicidal compounds 2-coumaranone and cyclo-(Pro-Phe) produced by *Lysinimonas* sp. M4 significantly inhibited the PWN egg hatching by 59.92% and 50.77%, respectively, at 0.1 mM concentration (Sun et al., 2022). The root drenching of *L. pinisoli* DP2-30 FB substantially mitigated the PWD in pine seedlings treated with PWN with a control effect of 62.50%. The achieved control effect of *L. pinisoli* DP2-30 against PWD might be due to the direct nematicidal activity of *L. pinisoli* DP2-30 on PWN and the induction of host resistance against PWN infestation. Previous studies have demonstrated that beneficial rhizobacteria trigger plant defense against PWN and effectively reduce PWD, supporting our findings (Han et al., 2021; Sun et al., 2022; Li et al., 2023a). Although not the main goal, the growth-promoting effect of *L. pinisoli* DP2-30 on tobacco seedlings indicates its potential as a strong plant growth-promoting rhizobacterium (PGPR). The ability of *L. pinisoli* DP2-30 to serve as both a BCA and PGPR is highly advantageous for sustainable agriculture and forestry management. This finding is in accordance with previous reports that some rhizobacteria can play a dual role, promoting plant growth and providing protection from diseases simultaneously (Ayaz et al., 2021; Han et al., 2021; Ahmed et al., 2022b).

Analysis of the microbiome in *L. pinisoli* DP2-30 treated and untreated pine seedlings revealed notable differences in the proportions of bacterial and fungal communities in the roots and stems of the pine plants. These results align with prior findings that the use of BCAs alters the relative abundance of microbial communities (Han et al., 2021; Dai et al., 2023). The prominent bacterial phyla identified in all samples were Proteobacteria, Actinobacteriota, Chloroflexi, and Acidobacteriota, whereas the dominant fungal phyla were Ascomycota and Basidiomycota, corroborating the findings of previous researchers (Ponpandian et al., 2019; Zhang et al., 2022c). The proportion of Proteobacteria was notably greater in T1-R, T2-R, and T2-S samples than in the other samples, but Actinobacteriota, Chloroflexi, and Armatimonadota were more dominant in the CK-S samples. The Ascomycota phylum of fungi showed substantial variation across treatments and exhibited high relative abundance in the T1-R and T2-S samples. The results indicate a notable change in the microbial community composition in the treated samples compared with the untreated ones. This change involves an increase in the abundance of beneficial bacteria and fungi, specifically Proteobacteria and Ascomycota, which are recognized for their ability to decrease diseases (Mendes et al., 2011). *Lysobacter* and *Trichoderma*, which belong to Proteobacteria and Ascomycota, respectively, are widely used to prevent plants from diseases by producing specific secondary metabolites and volatile organic compounds (VOCs) (Khan et al., 2020; Liu et al., 2022; Dai et al., 2023; Xing et al., 2023). The notable increase in the proportion of Basidiomycota in the treated roots (T2-R) of the fungal communities is worth mentioning. This phylum includes various members, such as *Arbuscular mycorrhizae* and *Ectomycorrhizae*, which are known to establish mycorrhizal associations with plant roots. These associations improve the absorption of nutrients by plants and offer protection against pathogens (Sharma et al., 2014; Tripathi et al., 2017).

At the class and family levels Gammaproteobacteria and Alphaproteobacteria were the predominant bacterial classes and Rhodanobacteraceae and Burkholderiaceae were the most dominant families. The prevalence of Rhodanobacteraceae was notably higher in T2-S and T2-R samples than in the other samples, suggesting successful colonization by *L. pinisoli* DP2-30. Interestingly, the application of *L. pinisoli* DP2-30 (T2) resulted in a decrease in bacterial alpha diversity but an increase in fungal alpha diversity compared to control (CK) and PWN-infected (T1) plants. The reduction in bacterial alpha diversity in T2 might have occurred because the application of DP2-30 increased the relative abundance of Rhodanobacteraceae (to which *L. pinisoli* belongs). This finding is in accordance with the study of Li and colleagues, who reported that *B. subtilis* L1-21 enhanced its diversity in the host by successful colonization (Li et al., 2023b) and also Proteobacteria is fasting growing bacteria that prefers to grow under nutrient-rich conditions and healthy environment (Fierer et al., 2007). The PCoA and PERMANOVA results revealed distinct microbial community structures between the treated and control samples. These findings suggest BCAs may create a hostile environment for pathogens by promoting the abundance of beneficial microbes. Ahmed et al. (2022b) and Zhang et al (Zhang et al., 2022a), reported similar results, demonstrating that the application of beneficial microorganisms can effectively modify the structure of microbial communities, lowering the prevalence of decreases in agricultural crops. Sordariomycetes and Dothideomycetes were the most dominant classes in the fungal communities, whereas the most prevalent families were Nectriaceae and Diaporthaceae. Application of biocontrol strain DP2-30 significantly reduced the RA of family Diaporthaceae in the root and stem of pine seedling combine treated with PWN+DP2-30. Studies showed that members (*Diaporthe ampelina*, *D. eres*, and *D. foeniculina*) of Diaporthaceae found be pathogenic to grapevines and causes Phomopsis cane and leaf spot diseases (Fedele et al., 2024). This also suggest the antifungal potential of strain DP2-30, however future study is suggested to prove this claim.

Intra-kingdom co-occurrence network analysis revealed that the complexity of bacterial co-occurrence networks was reduced from the root to stem in both control (CK) and *L. pinisoli* DP2-30-treated (T2) plants compared to PWN-infected (T1) plants, where network complexity increased. These findings suggests that the populations of the bacterial ecological network increased following PWN infection. The decrease in bacterial network complexity in T2 plants suggests a more stable and resilient microbial community structure, which could help suppress diseases. Conversely, complexity of fungal co-occurrence network was increased from root to stem in the pine seedlings under different treatments (CK, T1, and T2), with the most complex network was observed in the stem of pine seedlings under treatment T2 (T2-S). This increased complexity in fungal networks may be due to the recruitment of more fungal communities that play a role in disease suppression. Previous studies revealed that bacterial communities nodes or network complexity significantly increased in the phyllosphere and rhizosphere of pine plants after PWN infection (Deng et al., 2021).

## 5 Conclusions

**Figure 9:**
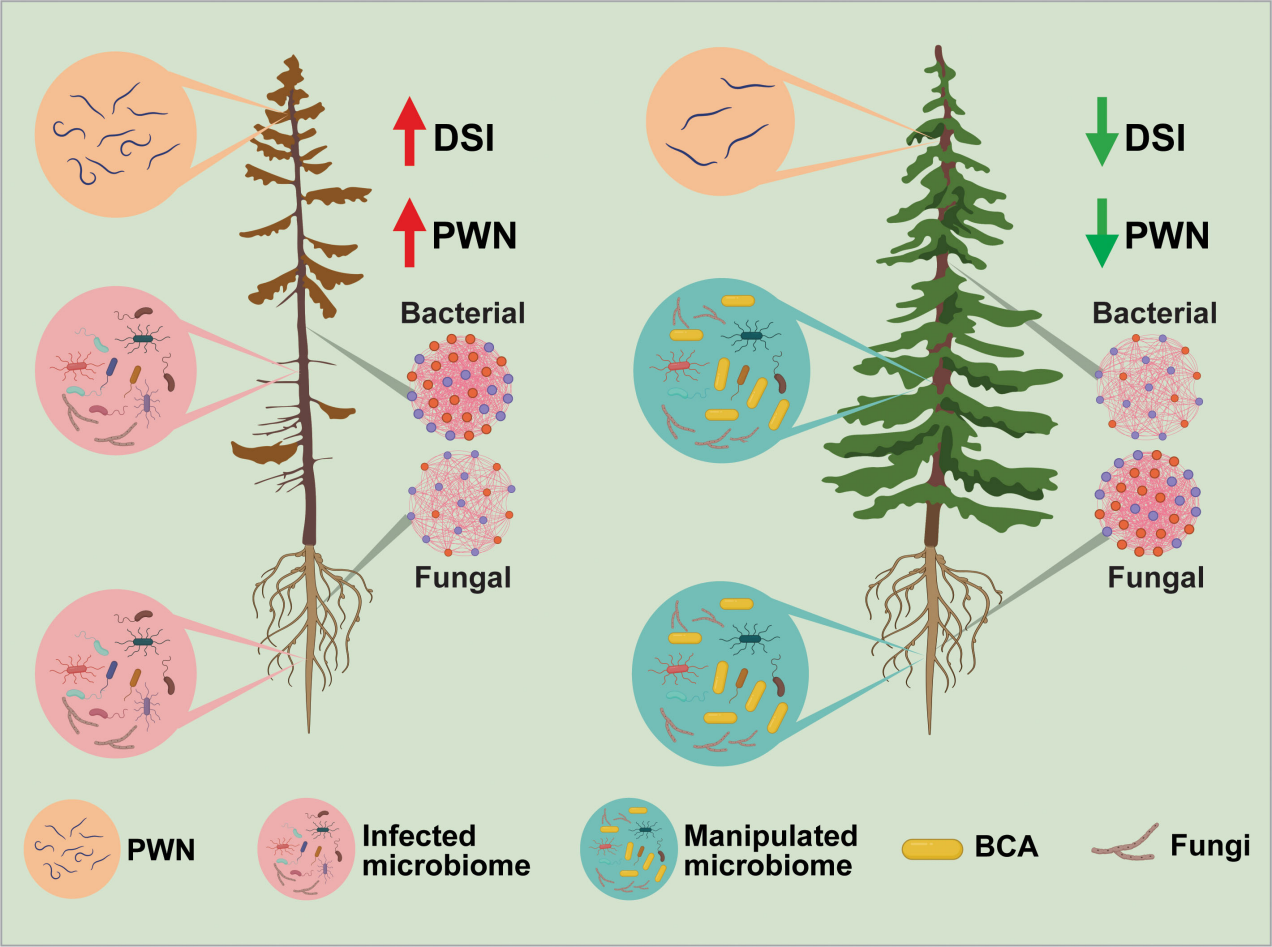
The concluding figure illustrates the effects of biocontrol agent (BCA) on the host microbiome and its role in reducing pine wilt disease. *Luteibacter pinisoli* DP2-30 (BCA) altered the composition of the host microbiome, promoting the growth of beneficial microorganisms and increasing microbial diversity, resulting in reduced PWD severity and healthier pine trees. The left side displays the pine tree subjected to the PWN, exhibiting a reduced abundance of beneficial microbes, a higher population of PWN, and higher disease incidence. The right side displays the pine tree treated with PWN+BCA, which showed an increase in beneficial microbes and an elevated microbial diversity, thus resulting in a healthier pine tree. The microbial networks depict the alterations in the diversity and interactions of microbial populations. The bacterial networks in the BCA-treated plants exhibited decreased complexity, whereas the fungal networks showed increased complexity, indicating a more stable and resilient microbial community structure.

In summary, we conclude that *Luteibacter pinisoli* DP2-30 isolated from the rhizosphere soil of *P. massoniana* demonstrated solid nematicidal activity against PWN, with a corrected mortality rate of 92.08%, and could be used as a promising biocontrol agent for the management of pine wilt disease. The optimization of fermentation conditions (pH 7 and 3 days of incubation at 28°C and 180 rpm) plays a role in maximizing (>95%) the nematicidal activity of strain DP2-30 against PWN. *L. pinisoli* DP2-30 fermentation broth and filtrates significantly inhibited PWN egg hatching, indicating that the metabolites produced during fermentation play a critical role in nematode suppression. Additionally, the application of *L. pinisoli* DP2-30 fermentation broth significantly reduced PWD severity in pine seedlings, with a control effect of 62.50%, demonstrating its efficacy in mitigating disease symptoms and improving plant health. A key finding of this study is the ability of *L. pinisoli* DP2-30 to reshape the pine seedling microbiome, potentially enhancing disease resistance. Intra-kingdom co-occurrence network analysis revealed reduced complexity in the bacterial networks but increased complexity in the fungal networks of the pine seedling combine treated with PWN+DP2-30, indicate a shift towards a community composition dominated by taxa that can effectively compete against PWN (**Figure 9**). This study also revealed the plant growth-promoting potential of *L. pinisoli* DP2-30, as it significantly enhanced the growth of tobacco seedlings. Overall, this study demonstrates the dual functionality of *L. pinisoli* DP2-30 as both a biocontrol agent and a plant growth-promoting bacterium, offering a sustainable and environmentally friendly strategy for managing PWD. However, future research will be carried out to optimize BCA formulations, clarify the specific underlying mechanisms of host-microbe interactions, and conduct field trials to validate the biocontrol efficacy of *L. pinisoli* DP2-30 under natural conditions.

## Data Availability

The datasets presented in this study can be found in online repositories. The names of the repository/repositories and accession number(s) can be found below: https://www.ncbi.nlm.nih.gov/genbank/, PRJNA1132778.
