# Supplementary material for: Evaluation the role of Luteibacter pinisoli DP2-30 in mitigating pine wilt disease caused by Bursaphelenchus xylophilus through modulation of host microbiome
Source: Front Plant Sci. 2025 Mar 5;16:1515506. doi: 10.3389/fpls.2025.1515506 (PMC11921891; doi:10.3389/fpls.2025.1515506)
Supplement: Supplementary file 1 [file DataSheet1.pdf]

Evaluation the role of *Luteibacter pinisoli* DP2-30 in mitigating pine wilt disease caused by *Bursaphelenchus xylophilus* through modulation of host microbiome

Waqar Ahmed[1], Wenhua Ye[1], Jidong Pan[1], Songsong Liu[1], Wenxia Ji[1], Shun Zhou[1], Fusheng Wang[1], Zhiguang Li[1], Mohamed Mohany[2], and Xinrong Wang[1,*]

[1] Guangdong Province Key Laboratory of Microbial Signals and Disease Control, College of Plant Protection, South China Agricultural University, Guangzhou 510642, Guangdong, China

[2] Department of Pharmacology and Toxicology, College of Pharmacy, King Saud University, Riyadh 11451, Saudi Arabia

***Corresponding author**

Name: Prof. Dr. Xinrong Wang

e-mail: xinrongw@scau.edu.cn

Table S1. Effect of *Luteibacter pinisoli* DP2-30 on tobacco seed germination and seedling growth.

Treatments	Germination rate (%)	Mean stem length (cm)	Mean root length (cm)	Fresh weight (g)
FB	93.33±2.74a	0.692±0.127a	1.642±0.084a	0.184±0.017a
FFs	91.11±1.57a	0.670±0.068a	1.505±0.057a	0.173±0.009a
CK-1	90.00±2.72a	0.600±0.139a	0.938±0.075b	0.116±0.015b
CK-2	90.00±2.62a	0.614±0.089a	0.942±0.123b	0.114±0.021b

Here: CK-1; seeds treated with NB, CK-2; seeds treated with sdH2O, FB; seeds treated with *L. pinisoli* DP2-30 fermentation broth and FFs; seeds treated with *L. pinisoli* DP2-30 fermentation filtrates. Data are present as ±std of three biological replicates. According to Duncan's test, different letters within column indicate significant differences at $p < 0.05$ level.

Table S2. Data preprocessing, statistics, and quality control of raw data obtained from different samples by amplification of V5-V7 variable region of bacterial 16S rRNA gene.

Samples	Raw Reads	Clean Reads	Raw Tags	Clean Tags	Chimera	Effective Tags	Effective Ratio (%)	Taxon Tags	Max length (bp)	OTUs
CK-R-1	124505	123964	118239	116865	28142	88723	71.26	58204	465	1146
CK-R-2	121844	121318	112631	111352	26949	84403	69.27	49797	465	1261
CK-R-3	132956	132193	122577	121066	27523	93543	70.36	59494	474	1276
CK-R-4	125697	125059	116468	115134	22942	92192	73.34	67141	472	1184
CK-R-5	132877	132341	126028	124709	28353	96356	72.52	64503	474	1199
CK-R-6	125765	125181	118200	116768	26685	90083	71.63	53401	474	1332
T1-R-1	135226	134695	128766	127255	24383	102872	76.07	85307	465	852
T1-R-2	129030	128462	122293	120902	28366	92536	71.72	69104	466	944
T1-R-3	133090	132511	127153	125773	29563	96210	72.29	74787	465	847
T1-R-4	122776	122206	116805	115435	27072	88363	71.97	63748	465	905
T1-R-5	131127	130413	123579	122136	23866	98270	74.94	79907	463	809
T1-R-6	120060	119450	113758	112517	20820	91697	76.38	77518	465	799
T2-R-1	136226	135596	129270	127711	29059	98652	72.42	80125	465	886
T2-R-2	128785	128209	122984	121548	28382	93166	72.34	73507	465	942
T2-R-3	135404	134823	128954	127300	23245	104055	76.85	91327	465	832
T2-R-4	132336	131757	126583	125019	26524	98495	74.43	83404	465	835
T2-R-5	128438	127908	118675	117320	27380	89940	70.03	69331	465	964
T2-R-6	124924	124359	114574	113253	27349	85904	68.77	64719	457	884
CK-S-1	135758	135103	105176	103867	24503	79364	58.46	53969	474	747
CK-S-2	131546	130913	108375	107131	19693	87438	66.47	69789	468	711
CK-S-3	137277	136733	118131	116940	22423	94517	68.85	72841	474	742
CK-S-4	122336	121787	103533	102319	20490	81829	66.89	59652	474	765
CK-S-5	131946	131382	115614	114287	22259	92028	69.75	71801	473	706
CK-S-6	135426	134850	109005	107684	20448	87236	64.42	69585	474	668
T1-S-1	121384	120798	114395	113101	16803	96298	79.33	84444	465	708
T1-S-2	123841	123221	113311	112086	22261	89825	72.53	71699	474	723
T1-S-3	132834	132151	118020	116584	24174	92410	69.57	73372	474	729
T1-S-4	109465	109108	102265	101343	24324	77019	70.36	60261	465	736
T1-S-5	80055	79798	74228	73578	19084	54494	68.07	39590	465	737
T1-S-6	92731	92452	85475	84664	21960	62704	67.62	43046	474	801
T2-S-1	137554	136938	128956	127375	19379	107996	78.51	97453	468	559
T2-S-2	129679	129087	119693	118297	18426	99871	77.01	89192	474	554
T2-S-3	132349	131777	125167	123536	17410	106126	80.19	97891	465	537
T2-S-4	129863	129233	121136	119694	16721	102973	79.29	93512	474	541
T2-S-5	120083	119518	108809	107446	16591	90855	75.66	81940	465	504
T2-S-6	122240	121757	114327	113091	16940	96151	78.66	87809	465	544
**Sum**	4547433	4527051	4173153	4125086	840492	3284594	/	2583170	/	29909
**Ave**	126318	125751	115920.9	114585.7	23347	91239	/	71754	/	830

Here. Sample name: sample collected from different treatments, raw reads: original pair-ed reads, clean reads: high-quality pair-end reads obtained after QC filtering, raw tags: the number of original tags obtained by overlapping assembly, clean tags: the number of high-quality tags obtained after tags quality control, chimera: number of chimera tags detected during OTU clustering, effective tags: The number of high-quality tags after removing the chimera is the valid tag for subsequent analysis, effective ratio (%): the percentage of the number of high-quality tags, taxon tags: the number of tags with species notes, mam length (bp): length of the longest tag, and OTUs: the final number of OTUs. Here, CK; application of water as control, T1; application of PWNs, and T2; application of PWNs + *L. pinisoli. R = roots and S = stems*

Table S3. Data preprocessing, statistics, and quality control of raw data obtained from different samples by amplification of ITS1 variable region of fungal ITS gene.

Samples	Raw Reads	Clean Reads	Raw Tags	Clean Tags	Chimera	Effective Tags	Effective Ratio (%)	Taxon Tags	Max length	OTUs
CK-R-1	120062	120040	117389	116283	2271	114012	94.96	113250	476	392
CK-R-2	128810	128776	126061	124807	2016	122791	95.33	121846	464	434
CK-R-3	134808	134783	131741	130412	2071	128341	95.2	127638	447	402
CK-R-4	125188	125162	122960	121937	1757	120180	96	119604	440	398
CK-R-5	124241	124203	121826	120745	2145	118600	95.46	117819	475	398
CK-R-6	120764	120749	118094	116937	2772	114165	94.54	113353	475	406
CK-S-1	127700	127666	119545	118135	3373	114762	89.87	113957	476	588
CK-S-2	132940	132918	123722	121974	3023	118951	89.48	118208	474	605
CK-S-3	127785	127752	120354	118942	4084	114858	89.88	113949	476	627
CK-S-4	125683	125643	118575	117000	2491	114509	91.11	113646	476	586
CK-S-5	135361	135322	124690	123201	3885	119316	88.15	118477	475	633
CK-S-6	120775	120738	109278	107728	2400	105328	87.21	104723	476	558
T1-R-1	122757	122734	119945	118981	2518	116463	94.87	115932	475	330
T1-R-2	136486	136452	133491	132383	2000	130383	95.53	129873	475	375
T1-R-3	133304	133273	129285	128089	1948	126141	94.63	125714	475	297
T1-R-4	132863	132841	130006	129007	2283	126724	95.38	126188	463	379
T1-R-5	124707	124595	121890	120880	1632	119248	95.62	118801	476	343
T1-R-6	133265	133240	130559	129232	3023	126209	94.71	125533	476	380
T1-S-1	127971	127949	124005	122894	1718	121176	94.69	120679	475	472
T1-S-2	121227	121208	117553	116487	1859	114628	94.56	114065	475	530
T1-S-3	126072	126048	122449	121187	2396	118791	94.22	118183	476	444
T1-S-4	120395	120359	117133	115985	1763	114222	94.87	113793	468	484
T1-S-5	122753	122734	118641	117526	1875	115651	94.21	115110	472	508
T1-S-6	126349	126320	121846	120653	1466	119187	94.33	118753	465	490
T2-R-1	88843	88832	87040	86239	4430	81809	92.08	81219	470	372
T2-R-2	131109	131087	128753	127693	1733	125960	96.07	125331	476	391
T2-R-3	123754	123731	120718	119632	1182	118450	95.71	118047	474	375
T2-R-4	128889	128877	125625	124640	1364	123276	95.65	122805	476	373
T2-R-5	132797	132755	130036	128938	1379	127559	96.06	127079	454	377
T2-R-6	121160	121129	118864	117628	1979	115649	95.45	115028	451	407
T2-S-1	100808	100788	95023	94029	3377	90652	89.93	89841	476	611
T2-S-2	124978	124958	119391	118124	3013	115111	92.11	114302	476	568
T2-S-3	124927	124907	119301	118159	3739	114420	91.59	113518	472	629
T2-S-4	128453	128408	122956	121698	3105	118593	92.32	117601	476	589
T2-S-5	127916	127841	120251	119010	2644	116366	90.97	115631	476	593
T2-S-6	137351	137320	129869	128602	2765	125837	91.62	125108	476	608
**Sum**	4523251	4522138	4358865	4315797	87479	4228318	/	4204604	/	16952
**Avg**	125645.9	125614.9	121079.6	119883.3	2429.972	117453.3	/	116794	/	470

Here. Sample name: sample collected from different treatments, raw reads: original pair-ed reads, clean reads: high-quality pair-end reads obtained after QC filtering, raw tags: the number of original tags obtained by overlapping assembly, clean tags: the number of high-quality tags obtained after tags quality control, chimera: number of chimera tags detected during OTU clustering, effective tags: The number of high-quality tags after removing the chimera is the valid tag for subsequent analysis, effective ratio (%): the percentage of the number of high-quality tags, taxon tags: the number of tags with species notes, mam length (bp): length of the longest tag, and OTUs: the final number of OTUs. Here, CK; application of water as control, T1; application of PWNs, and T2; application of PWNs + *L. pinisoli. R = roots and S = stems*

Table S4. PERMANOVA test for variance of bacterial and fungal communities among different samples based on Adonis.

Bacteria (16S rRNA; V5-V7)							
**Comparison**	**Df**	**Sums of Sqs**	**Mean Sqs**	**F-value**	**R^2**	**P-value**	**Sig**
CK-R-vs-T1-R	1	0.6382	0.6382	10.705	0.517	0.001	**
CK-R-vs-T2-R	1	1.123	1.123	26.9017	0.729	0.002	**
T1-R-vs-T2-R	1	1.1613	1.1613	29.4949	0.7468	0.004	**
CK-S-vs-T1-S	1	0.4727	0.4727	9.9342	0.4983	0.003	**
CK-S-vs-T2-S	1	1.8625	1.8625	143.1821	0.9347	0.003	**
T1-S-vs-T2-S	1	1.514	1.514	38.9055	0.7955	0.004	**
CK-R-vs-CK-S	1	1.9153	1.9153	45.7844	0.8207	0.002	**
T1-R-vs-T1-S	1	0.9363	0.9363	14.3231	0.5889	0.003	**
T2-R-vs-T2-S	1	0.2522	0.2522	19.5229	0.6613	0.003	**
CK-R-vs-T1-R-vs-T2-R	2	1.9484	0.9742	20.7659	0.7347	0.001	**
CK-S-vs-T1-S-vs-T2-S	2	2.5661	1.2831	38.6834	0.8376	0.001	**
CK-R-vs-T1-R-vs-T2-R-vs-CK-S-vs-T1-S-vs-T2-S	5	6.9667	1.3933	34.7982	0.8529	0.001	**
**Fungi (ITS1)**							
**Comparison**	**Df**	**Sums of Sqs**	**Mean Sqs**	**F-value**	**R^2**	**P-value**	**Sig**
CK-R-vs-T1-R	1	0.8722	0.8722	10.7183	0.5173	0.006	**
CK-R-vs-T2-R	1	0.6191	0.6191	6.1817	0.382	0.001	**
T1-R-vs-T2-R	1	0.602	0.602	5.2743	0.3453	0.003	**
CK-S-vs-T1-S	1	0.8165	0.8165	9.4224	0.4851	0.003	**
CK-S-vs-T2-S	1	0.3401	0.3401	6.172	0.3816	0.004	**
T1-S-vs-T2-S	1	0.7272	0.7272	8.0952	0.4474	0.005	**
CK-R-vs-CK-S	1	2.3165	2.3165	38.8286	0.7952	0.005	**
T1-R-vs-T1-S	1	1.0914	1.0914	10.0706	0.5018	0.001	**
T2-R-vs-T2-S	1	1.8805	1.8805	19.6699	0.663	0.001	**
CK-R-vs-T1-R-vs-T2-R	2	1.3955	0.6978	7.0799	0.4856	0.001	**
CK-S-vs-T1-S-vs-T2-S	2	1.2559	0.6279	8.1342	0.5203	0.001	**
CK-R-vs-T1-R-vs-T2-R-vs-CK-S-vs-T1-S-vs-T2-S	5	6.9736	1.3947	15.8711	0.7257	0.001	**

Here, CK; application of water as control, T1; application of PWNs, and T2; application of PWNs + *L. pinisoli.* R = roots and S = stems. Asterisks indicates significant differences at $**p < 0.01$ according to Adonis.

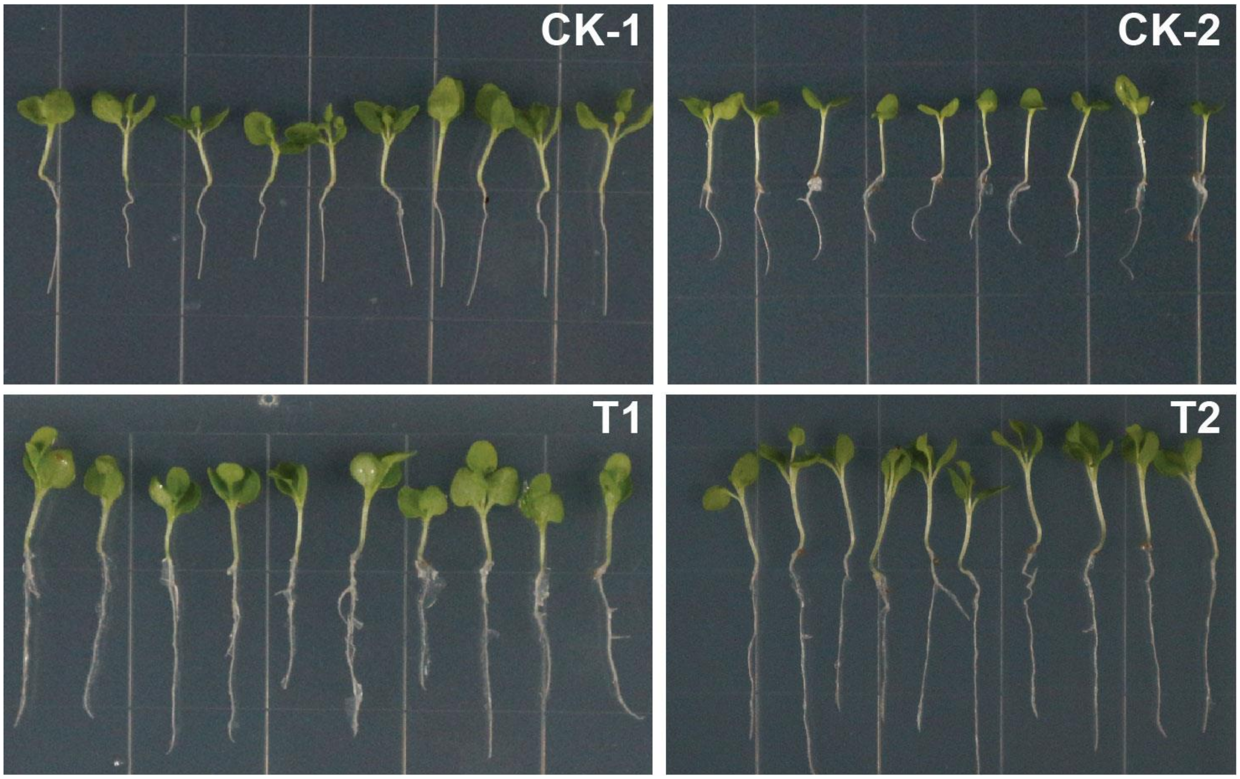

Figure S1. Plant growth-promoting potential of *Luteibacter pinisoli* DP2-30 on tobacco seedlings. CK-1; seeds treated with NB, CK-2; seeds treated with sdH2O, FB; seeds treated with *L. pinisoli* DP2-30 fermentation broth, and FFs; seeds treated with *L. pinisoli* DP2-30 fermentation filtrates.

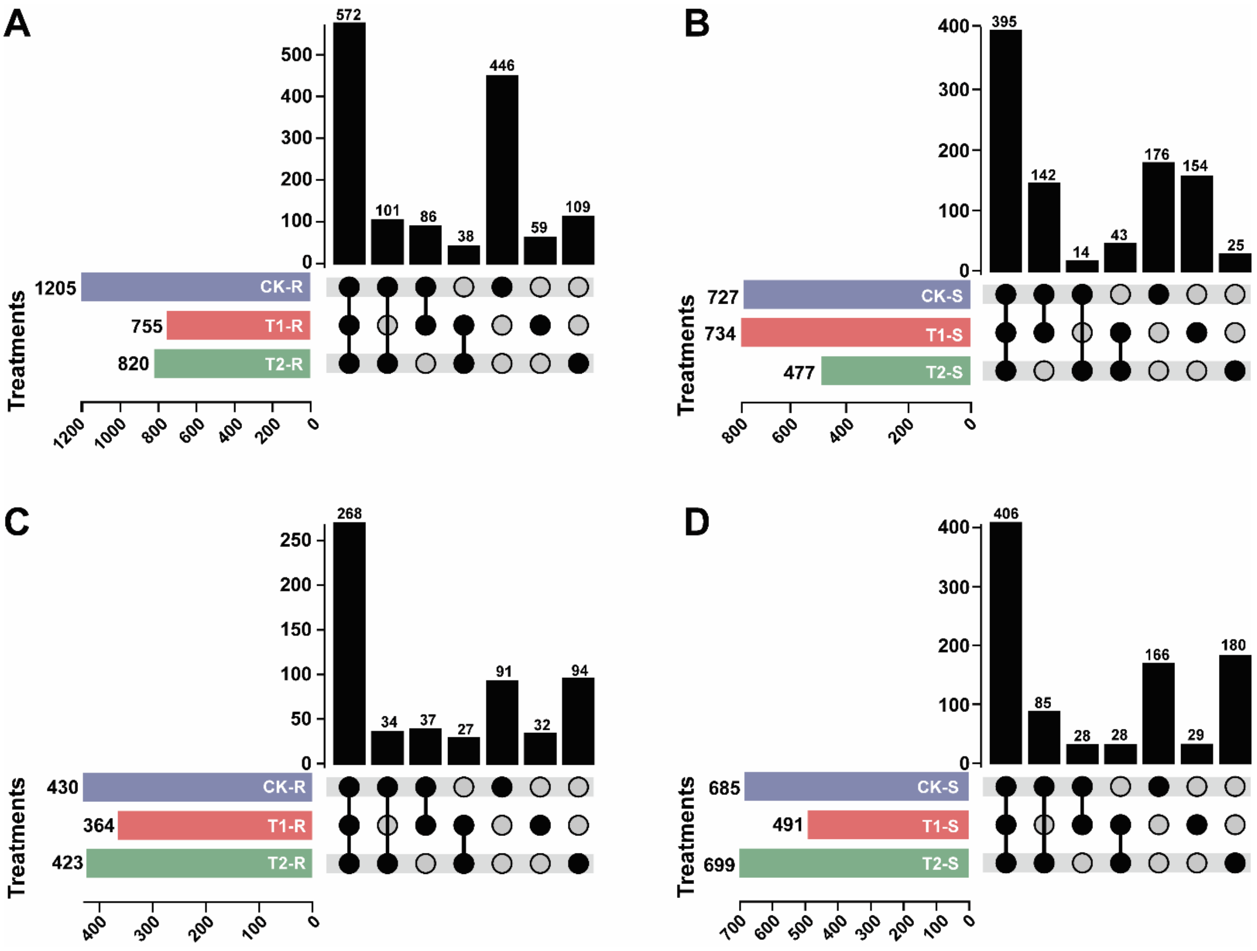

Figure S2. Upset plots showing the unique and common OTUs in different samples (roots and stems) among the treatments. Upset plots for bacterial (A-B) and fungal (C-D) OTUs. Here, CK; application of water as control, T1; application of PWNs, and T2; application of PWNs + *L. pinisoli.* R = roots and S = stems.
